# Investigation of reported small molecule P2Y6 receptor antagonists

**DOI:** 10.1007/s00044-026-03591-w

**Published:** 2026-07-11

**Authors:** Chianna Dane, André D.J. McKenzie, Andrew P. Montgomery, Jonathan Du, Eryn L. Werry, Michael Kassiou

**Affiliations:** 1 School of Chemistry, Faculty of Science, University of Sydney, Sydney, NSW 2006 Australia; 2 Sydney Pharmacy School, Faculty of Medicine and Health, University of Sydney, Sydney, NSW 2006 Australia

**Keywords:** P2Y6 receptor, GPCR, Antagonist, Small molecule, Validation

## Abstract

Interest in the P2Y6 receptor (P2Y6R) has increased substantially over the past decade, driven by evidence that pharmacological inhibition is beneficial in neurodegenerative disease models, inflammatory pathologies, and cancer. However, therapeutic targeting of the P2Y6R has remained a challenge due to a lack of available drug-like molecules. In 2023 and 2024, benzimidazole and quinoline chemotypes respectively, were reported as potent P2Y6R antagonists with pharmacological relevance in an inflammatory bowel disease model. In the present study positive and negative controls from each chemotype were selected, and their functional activity at hP2Y6R was assessed prior to chemical diversification. Contrary to the published literature, none of the antagonists exhibited functional activity in our Ca^2+^ mobilisation assay reported herein. These findings emphasise the ongoing challenges in reliably assessing pharmacological activity at the hP2Y6R and highlight a key barrier to progression of the field.

**Supplementary Information:**

The online version contains supplementary material available at https://doi.org/10.1007/s00044-026-03591-w.

**Figure:**
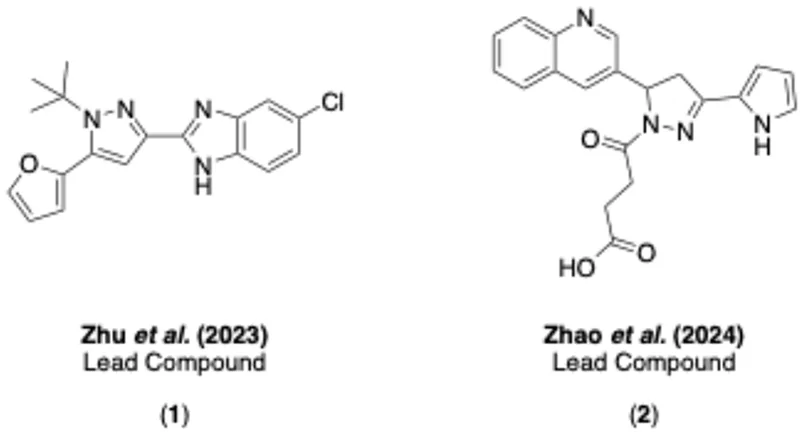

## Introduction

The P2Y6 receptor (P2Y6R) is a G_q_–protein-coupled receptor, expressed on multiple different cell types in the peripheral and central nervous systems [1]. Owing to its broad involvement in physiological and pathological processes, the P2Y6R influences cellular behaviour in immune as well as non-immune cells [1]. Upon activation by extracellular uridine diphosphate (UDP), the P2Y6R stimulates production of inositol triphosphate (IP3) and initiates the phospholipase C (PLC) signalling cascade, leading to a sustained rise in intracellular Ca²⁺ levels [2, 3]. This, in turn, promotes the production and release of pro-inflammatory cytokines and chemokines [3]. Additionally, the P2Y6R acts as a sensor for phagocytosis, underscoring its key role in inflammatory responses throughout the peripheral and central nervous systems [4, 5].

**Fig. 1:**
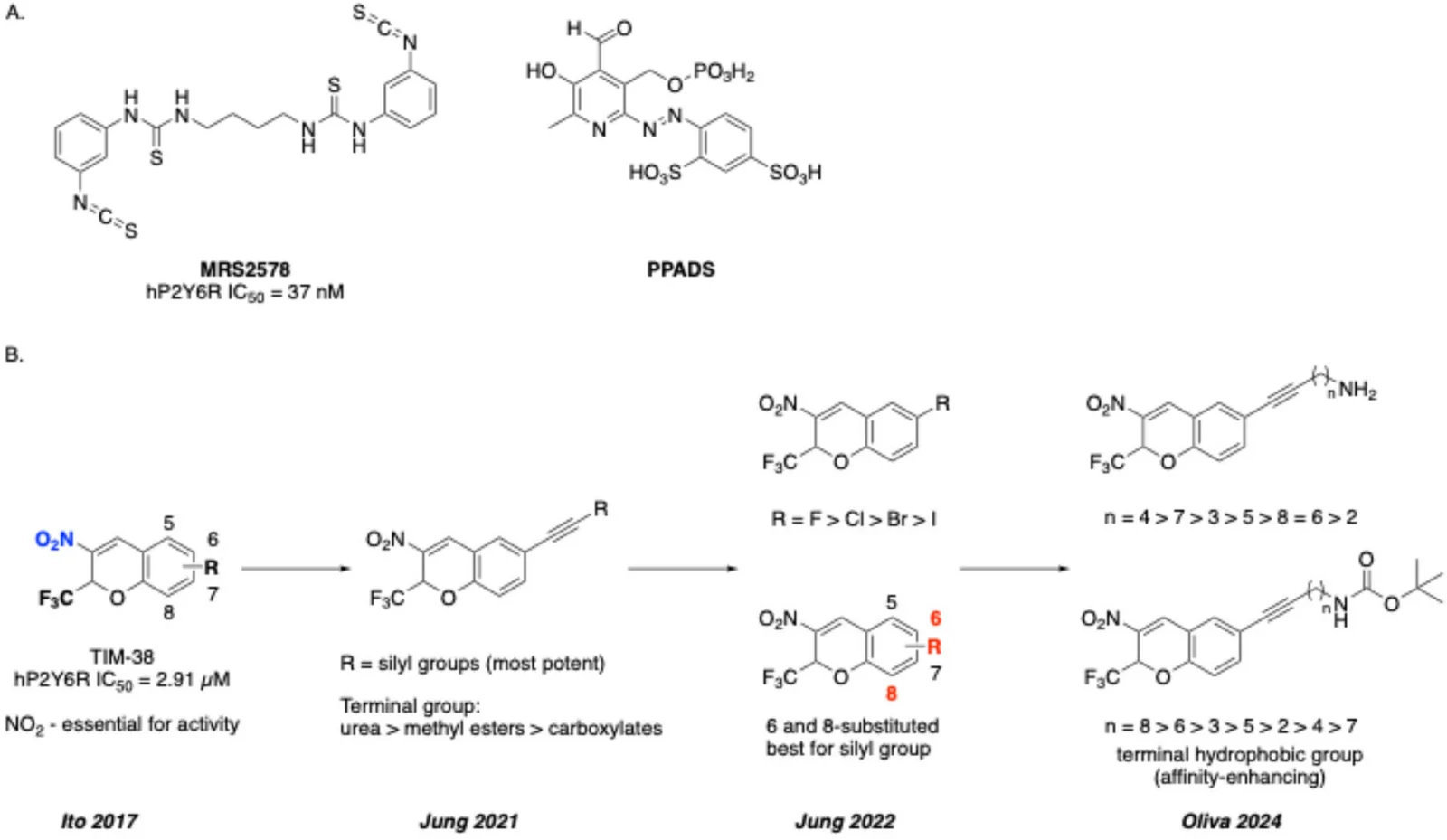
Select P2Y6R antagonists reported in literature. (**a**) Selective P2Y6R antagonist, MRS2578 and non-selective P2 antagonist, PPADS, are commonly utilised to investigate the pharmacological effect of inhibiting the P2Y6R. (**b**) Summary of the SAR conducted by the Jacobson group on the 2*H*-Chromene P2Y6R antagonist scaffold

Although targeting the P2Y6R therapeutically is increasingly supported across diverse pathologies, including neurodegeneration and cancer, progress remains limited by the lack of translatable drug-like inhibitors [3–10]. Currently, the gold standard for in vitro and in vivo studies is the isocyanato-substituted dimeric thiourea derivative developed by the Jacobson group, MRS2578 (Fig. 1A) [5, 8, 9, 11–15]. While valuable for interrogating P2Y6R pharmacology, it cannot be translated clinically due to its low water solubility and high reactivity [1, 11, 16–18].

The 2*H*-chromene analogues (Fig. 1B) reported by Jacobson and colleagues mark the most extensive structure activity relationship (SAR) analysis of a P2Y6R antagonist chemotype to date [19–22]. Consisting of a chromene core with an ethynyl-linked substitution on the unsaturated phenyl ring, this chemotype has shown high nanomolar activity at the human P2Y6R [19–22]. Key structural pharmacophores, i.e. the nitro group, have been shown to be essential for activity however the series displayed a largely flat SAR profile, with no clear preference for positioning, chain length and terminal functionality of the ethynyl-linked substituent [19–22]. Furthermore, the 2*H*-chromene analogues have been reported to have off-target inhibition at adrenergic and dopamine receptors, further reinforcing the demand for novel P2Y6R antagonists scaffolds [22].

**Fig. 2:**
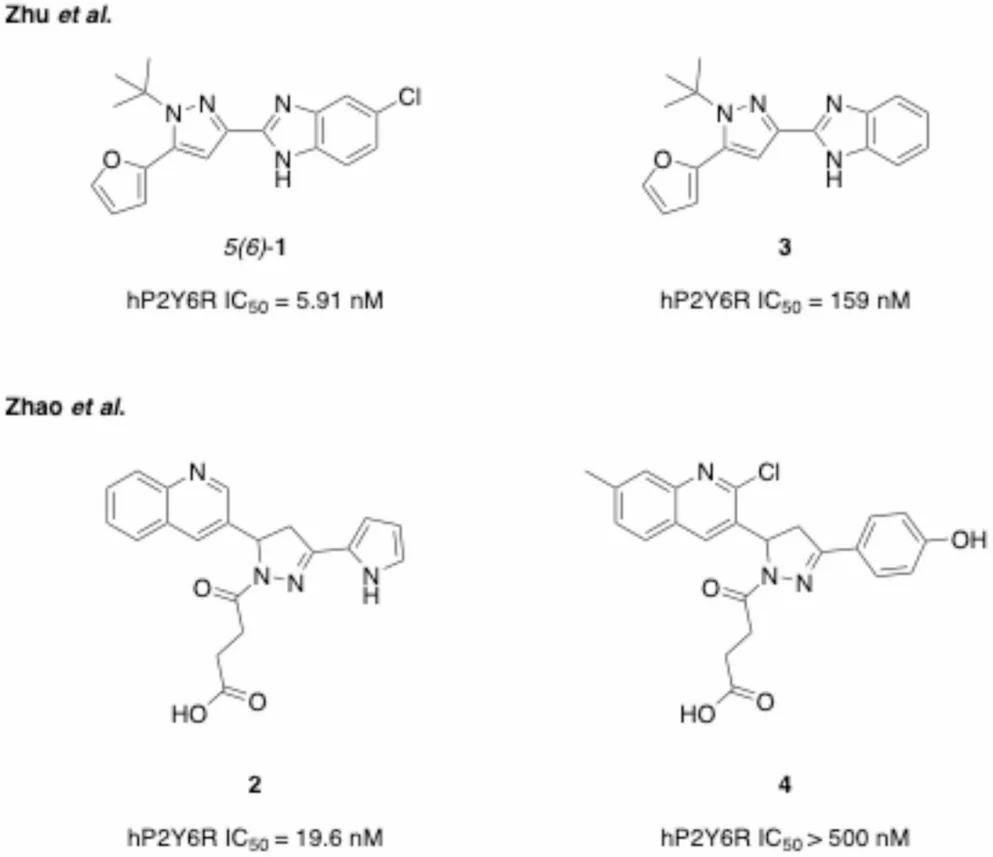
Positive (*5(6)-***1** and **2**) and negative (**3** and **4**) control compounds selected from the benzimidazole (Zhu et al., (2023)) and quinoline (Zhao et al., (2024)) scaffolds for validation. Data extracted from Zhu, Y. et al., 2023 (*5(6)-***1**, **3**) and Zhao, Y. et al., 2024 (**2**, **4**)

The need for novel P2Y6R antagonist chemotypes is supported by the recent publications from Zhu et al. (2023) and Zhao et al. (2024) [23, 24]. These studies described a series of pyrazole-benzimidazole (**1**) and pyrazoline-quinoline (**2**) compounds respectively, identified through a structure-based virtual screening (SBVS) approach (Fig. 2) [23, 24]. The initial pyrazole library presented by Zhu et al. (2023) was preceded by two patents, containing a total of 22 analogues with heteroaromatic variations to both the 5-membered and 5,6-membered rings [25, 26]. Building off this work, Zhao and coworkers published pyrazoline-quinoline derivatives in 2024 [24]. Utilising an identical SBVS approach, with Zhu’s P2Y6R homology model as validation, Zhao and coworkers identified compound **2** as a potent P2Y6R antagonist with biological relevance in an inflammatory bowel disease model [24]. The low nanomolar potency reported for compounds **1** and **2** represented an exciting progression towards the development of P2Y6R antagonists [23, 24]. In the absence of P2Y6R structural data, the two chemotypes provide an excellent framework for future derivatisation, thereby expanding the current SAR for potent and selective P2Y6R antagonists. Although, to achieve this, initial validation of the literature compounds was required. We aimed to synthesise lead compounds **1** and **2**, and two compounds deemed ‘inactive’ (**3** and **4**), and evaluate their activity at the hP2Y6R.

## Results and discussion

### Synthesis

**Scheme 1:**
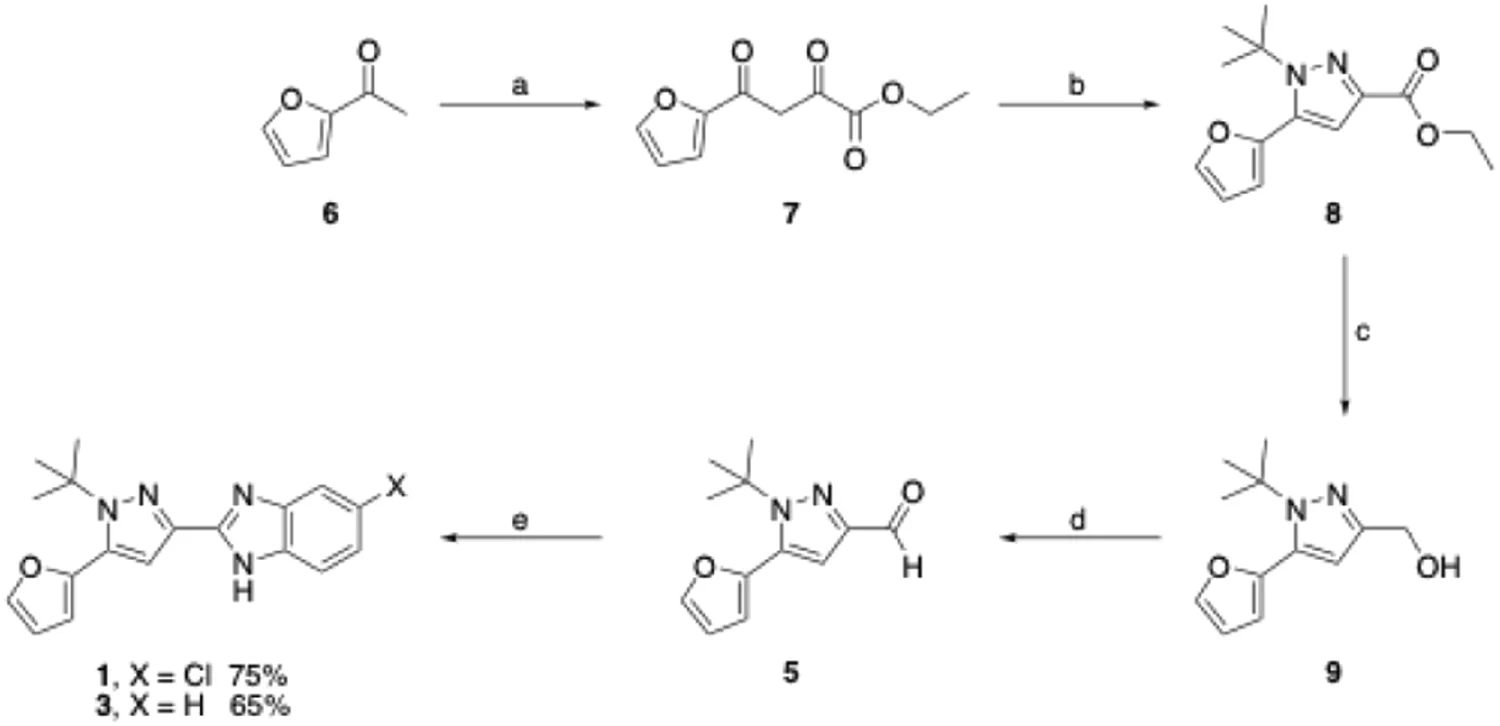
Synthesis of benzimidazole validation compounds **1** and **3**. *Reagents and Conditions* (a) diethyl oxalate, *t*-BuOK, ethylene glycol dimethyl ether, THF, 0 °C – rt, 1 h, 63–83% (b) *tert*-butylhydrazine hydrochloride, EtOH, rt, 8 h, 94% (c) LiAlH_4_, THF, reflux, 2 h, 86–96% (d) DMP, CH_2_Cl_2_, rt, 2 h, 60–94% (e) 4-chlorobenzene-1,2-diamine or benzene-1,2-diamine, NaHSO_3_, EtOH: H_2_O (2:1), reflux, 2 h

The benzimidazole (**1** and **3**) analogues were synthesised *via* the late-stage diversification of the aldehyde intermediate **5** (Scheme 1). An initial Claisen condensation between 2-acetylfuran (**6**) and diethyl oxalate yielded the 1,3-dicarbonilic intermediate **7**. The 1,3-dicarbonyl **7** was then treated with *tert*-butyl hydrazine hydrochloride to form the corresponding 1,3,5-pyrazole **8**. Subsequent reduction and oxidation of the ester (*via* the primary alcohol **9**) gave the key aldehyde intermediate **5**. Compound **5** was reacted with the appropriate diamine or 2-aminophenol to yield the target compounds **1** and **3**.

**Scheme 2:**
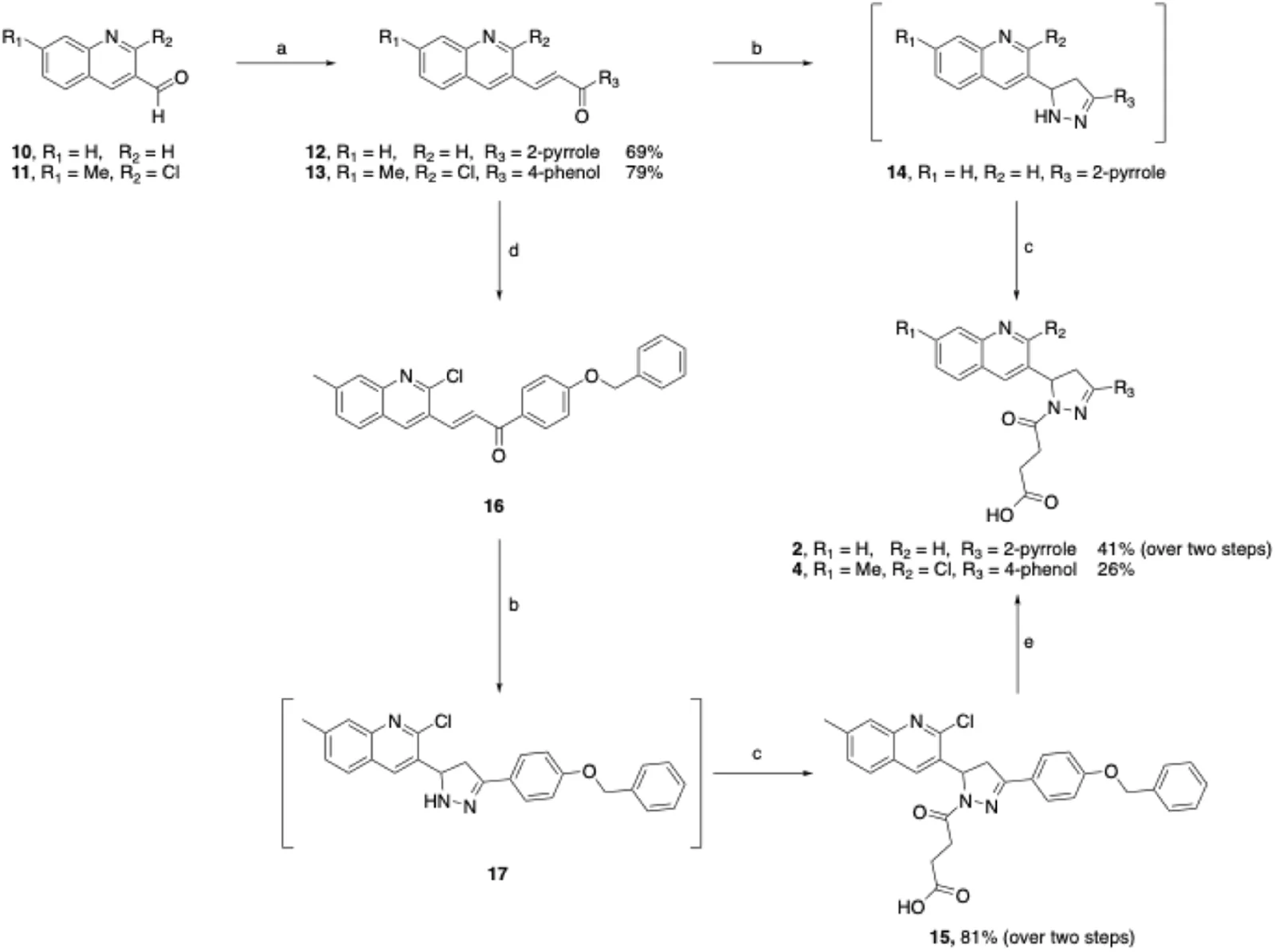
Synthesis of quinoline-dihydropyrazole validation compounds. *Reagents and Conditions* (a) appropriate acetyl compound, 2.5 M NaOH, EtOH, rt, 1 h (b) hydrazine monohydrate, EtOH, reflux, 1 h, not isolated (c) succinic anhydride, CH_2_Cl_2_, reflux, 2 h (d) benzyl bromide, NaH, DMF: THF (2:1), rt, 3 h, 90% (e) 1 M BBr_3_, CH_2_Cl_2_, 0 °C – rt, 1 h, 26% (f) Ac_2_O, 80 °C, 3 h, 82%

The quinoline derived analogues (**2–4**) were synthesised *via* an adapted route to that reported by Zhao et al. (2024), as presented in Scheme 2 [24]. The respective quinoline carboxaldehyde (**10** and **11**) was reacted with acetyl compounds under basic conditions to give the α,β-unsaturated diketones **12** and **13**. Compound **12** was then reacted with hydrazine monohydrate in ethanol to furnish the dihydropyrazole intermediate **14**. Refluxing in ethanol with succinic anhydride obtained the target compound **2** in moderate yields. Compound **4** was obtained from the benzyl protected analogue **15**. An analogous synthetic scheme was then utilised to achieve **15**.

### In silico

A template-based P2Y6R homology model created by Zhu et al. (2023) was foundational in both SBVS pipelines used to identify compounds **1** and **2** [23, 24]. Therefore, prior to further library development, redocking experiments of compounds **1–4** were conducted in the template-homology model to validate the in silico study. Both tautomeric and enantiomeric forms of **1** and **2** respectively, were assessed within the in silico model, as no discussion regarding the isolated derivative was reported in the corresponding publications. Redocking experiments, therefore, refer to the compound form presented in the corresponding figures [23, 24]. MRS2578 was not included in the ligand library due to its covalent binding nature and therefore potential to produce misleading docking results [27, 28].

**Fig. 3:**
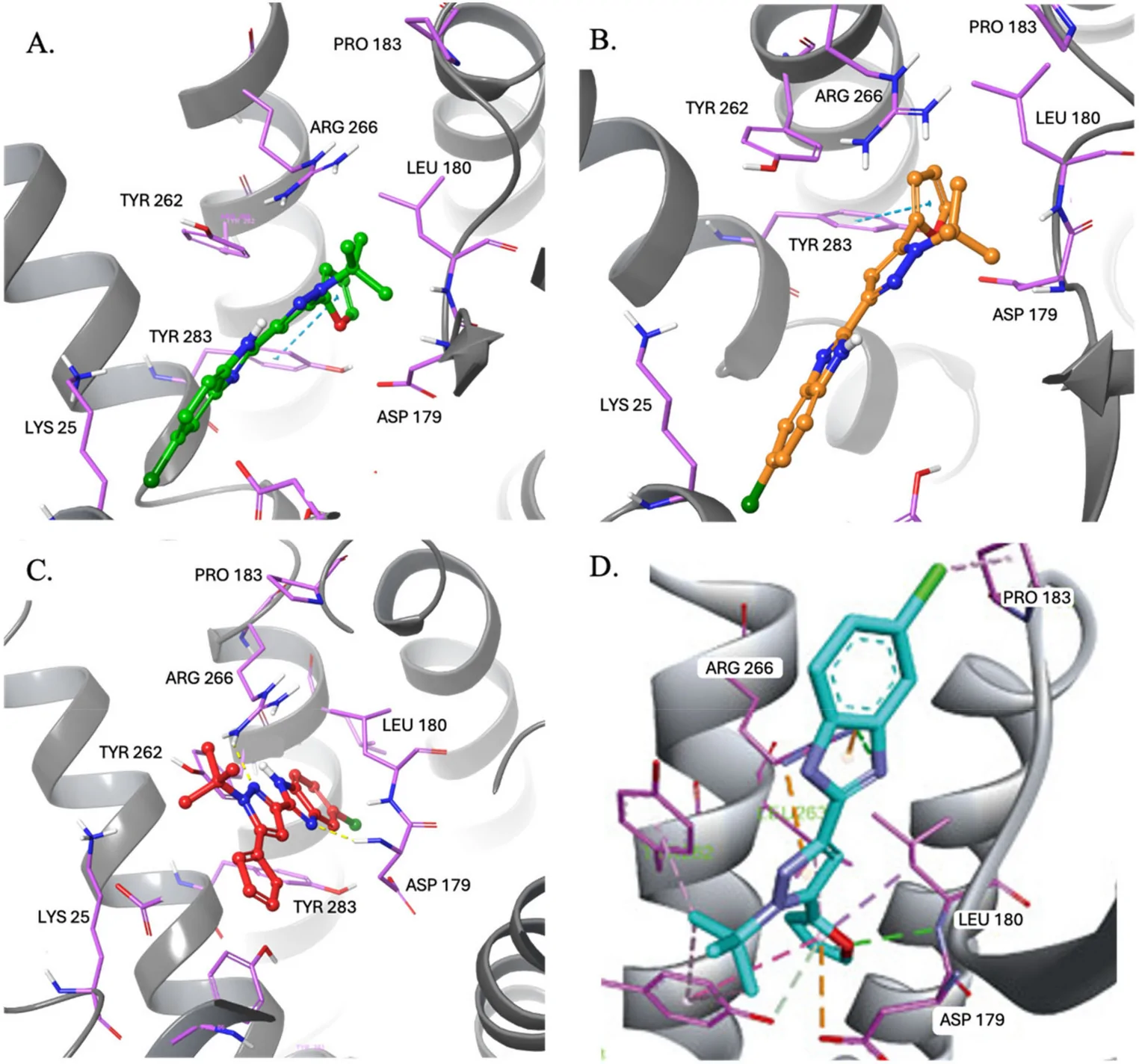
Re-docking of *5* − **1** in the P2Y6R templated homology model under RTD OPLS4 (**A**, green), RTD OPLS2005 (**B**, orange) and IFD OPLS4 (**C**, red) methods, in comparison to the results published by Zhu et al. (2023) (**D**). Reprinted (adapted) with permission from *J. Med. Chem.* 2023, 66, 9, 6315–6332. DOI: 10.102/acs.jmedchem.3c00210. Copyright 2023 American Chemical Society

Redocking experiments of the benzimidazole ligand *5 −* **1** were conducted under rigid-target (RTD) (Fig. 3A and B), induced-fit (IFD) (Fig. 3C) and varied force field (OPLS2005 and OPLS4) to mirror the methodology reported by Zhu et al. and reduce bias, respectively [23]. Methodology regarding molecular docking was omitted by Zhao et al. [24]. All redocking experiments were unable to recreate the pose presented in Zhu et al. (2023) (Fig. 3D) [23]. The quinoline lead structure *(R)-***2** successfully replicated the characteristic hydrogen bonding interaction between the carboxylate ion and Lys25 and Lys284 under RTD conditions, however ultimately failed redocking experiments with no pose able to replicate all reported interactions [24].

Although an improvement in sequence identity to the previously reported rhodopsin-based homology models of the P2Y6 receptor, significant limitations were identified in the model reported by Zhu et al. [29–32]. AI-based methods such as AlphaFold (AF) have been heralded as a more accurate method of predicting unknown protein structures to template-based methods, due to their utilisation of bioinformatic and physical information [33, 34]. Therefore, utilising localColabFold we established an AF homology model to further probe the validity of the template homology model proposed by Zhu et al. [33, 34]. All molecular docking experiments were conducted in an identical manner to that of the template-homology model, keeping the defined ligand binding site constant. Removing disordered *C-* and *N*-terminal loops, structural alignment of the two protein backbones reported an encouraging RMSD value of 1.8 Å (Supplementary Fig. 1). However, the homology model developed by Zhu et al. (2023), is templated off the P2Y1 receptor (P2Y1R) with an allosteric ligand bound [23]. With only 28% shared amino acid sequence identity, the P2Y6R and P2Y1R fail regular alignment requirements for a successful homology model [35, 36]. Low sequence identity (< 30%) can lead to false folding assignments due to alignment errors resulting in some regions of the protein not being properly modelled [35, 37]. Moreover, the accuracy of the current homology model is further compromised through the utilisation of an allosteric ligand to define the receptor grid. This is particularly relevant in the case of the P2Y1 receptor, which has been identified to contain a unique hydrophobic region when compared to other P2Y subtypes [38].

**Fig. 4:**
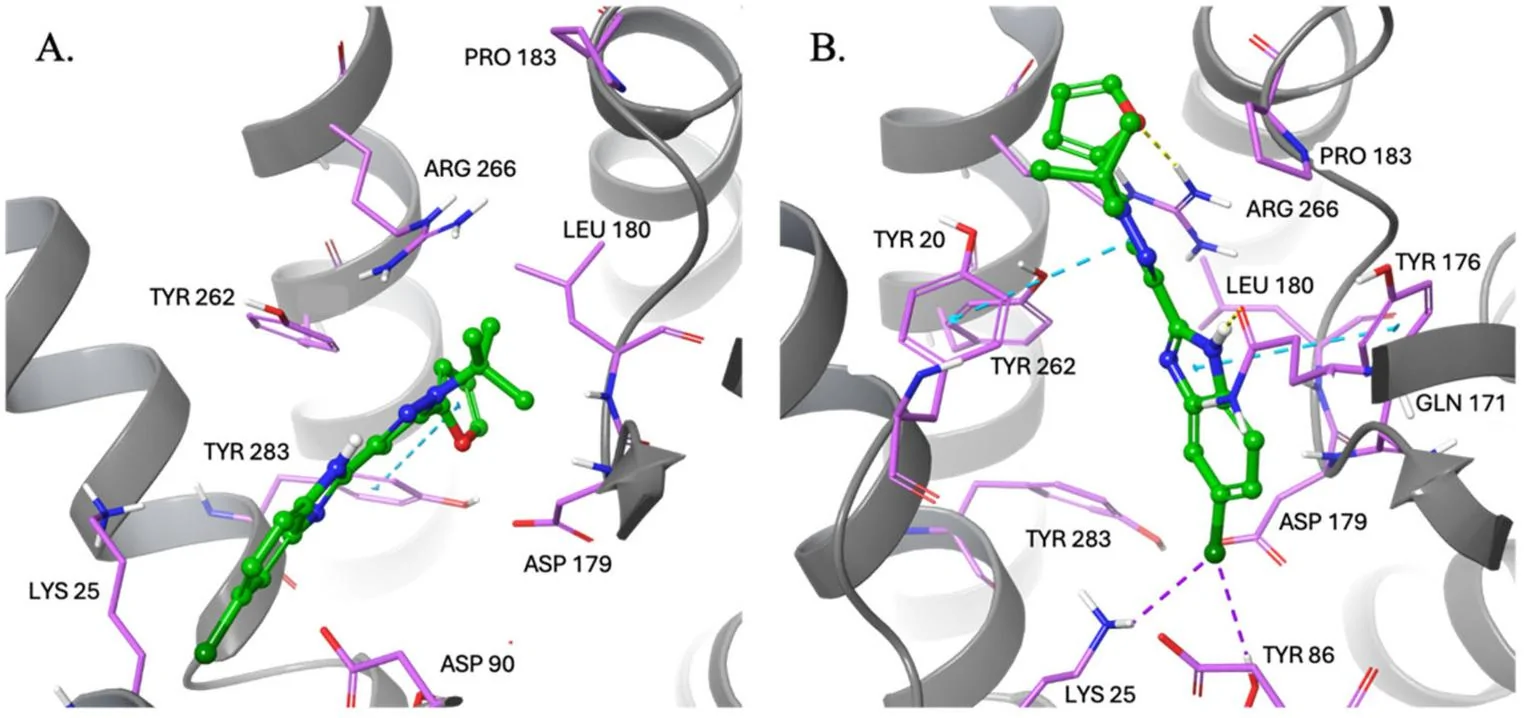
*5* − **1** displayed distinct binding modes in the template (**A**) and AlphaFold (**B**) homology models. Key binding interactions — e.g. hydrogen-bonds (yellow), π-π stacking (blue), arene-H interaction (cyan), halogen bond (purple) — denoted by the dotted coloured lines. Steric clashes with the surrounding protein are predicted in both models for the *tert*-butyl motif

Compound *5* − **1** demonstrated a distinct binding mode in the AF homology model as opposed to the template homology model (Fig. 4). The orientation of *5* − **1** remained consistent between the two models with the benzimidazole motif pointing outwards from the receptor. However, within the AF homology model, favourable π-π stacking interactions between the benzimidazole and pyrazole, with Tyr178 and Tyr20 respectively were observed (Fig. 4B). Furthermore, stable interactions were also formed with Lys25, Tyr86, Gln171 and Arg266, resulting in an improved docking score to its homology counterpart (Supplementary Table 2).

**Fig. 5:**
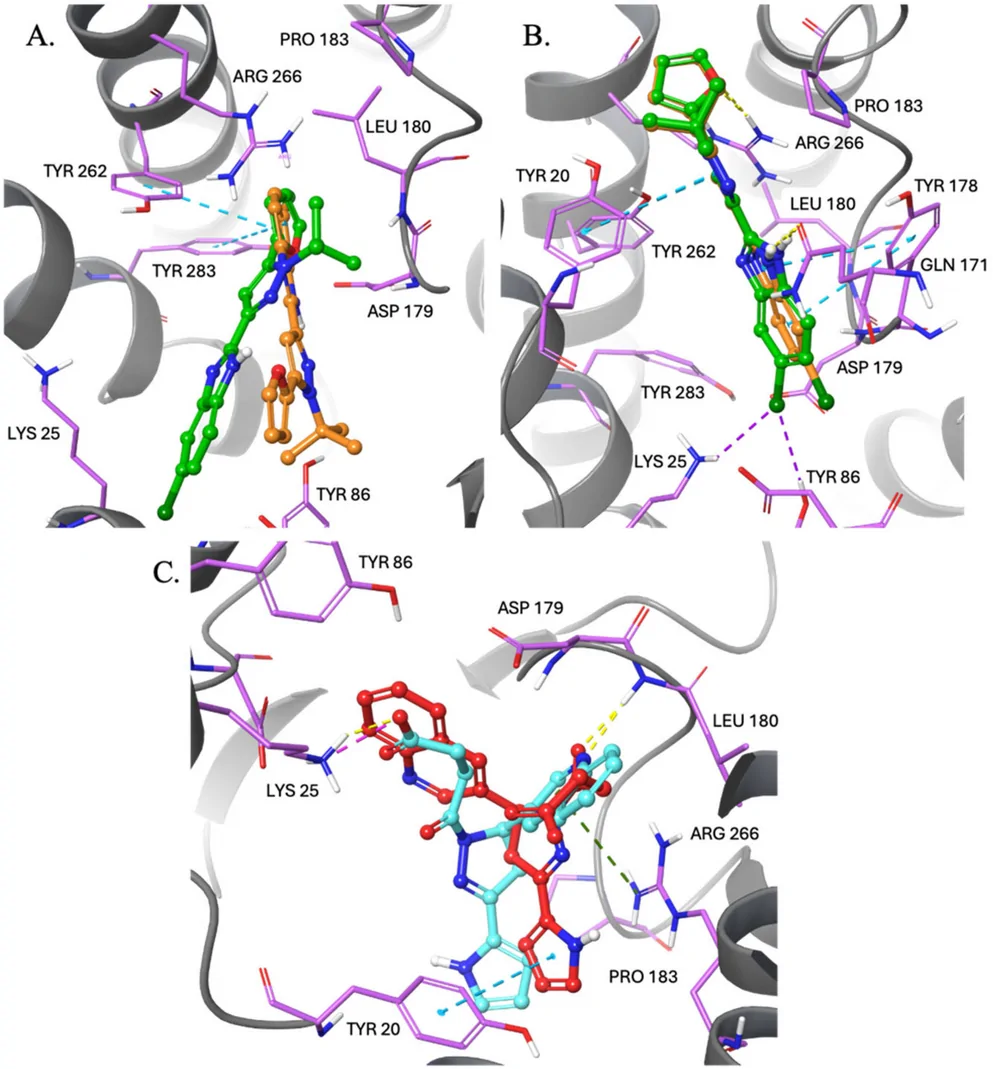
Comparison of predicted binding modes of **1** tautomers in the template (**A**) and AlphaFold (**B**) homology models. *5* − **1** is shown in green, and *6* − **1** is displayed in orange. The chiral centre of **2** (**C**) causes variation in binding interaction between enantiomers (*(R)*: cyan, *(S)*: red)

Interestingly, the tautomers of compound *5(6)-***1** were predicted to adopt opposite orientations within the template homology model (Fig. 5A). Whereas the two tautomeric forms are observed to be generally overlayed with consistent key interactions in the AF homology model (Fig. 5B). Similarly, the two enantiomers of the dihydropyrazole quinoline compound **2** produced different docking predictions. No trend was observed to predict the orientation of the dihydropyrazole motifs in either homology model. In the AF homology model, the enantiomers of **2** described a common π-π stacking interaction between Tyr20, with subsequent variation in binding interactions clearly ascribed to the chiral center (Fig. 5C). Although, no common interaction was observed when **2** was docked in the template homology model. Analysis of all isomeric forms showed that none successfully reproduced the reported docking interactions. Although both molecular models indicate that differences in tautomeric or enantiomeric binding modes do not substantially alter the compounds’ ability to bind to the P2Y6R (as suggested by similar docking scores), these differences may still influence functional activity (Supplementary 2). However, it must be acknowledged that the similar docking scores for the isomeric forms may be a pure coincidence due to the inherent limitations for docking methods to accurately predict binding energies between isomeric forms of compounds [39]. Therefore, the focus must be weighted heavily towards the predicted binding poses and interactions, and ultimately must be assessed *via in vitro* assays [39].

### In vitro

**Fig. 6:**
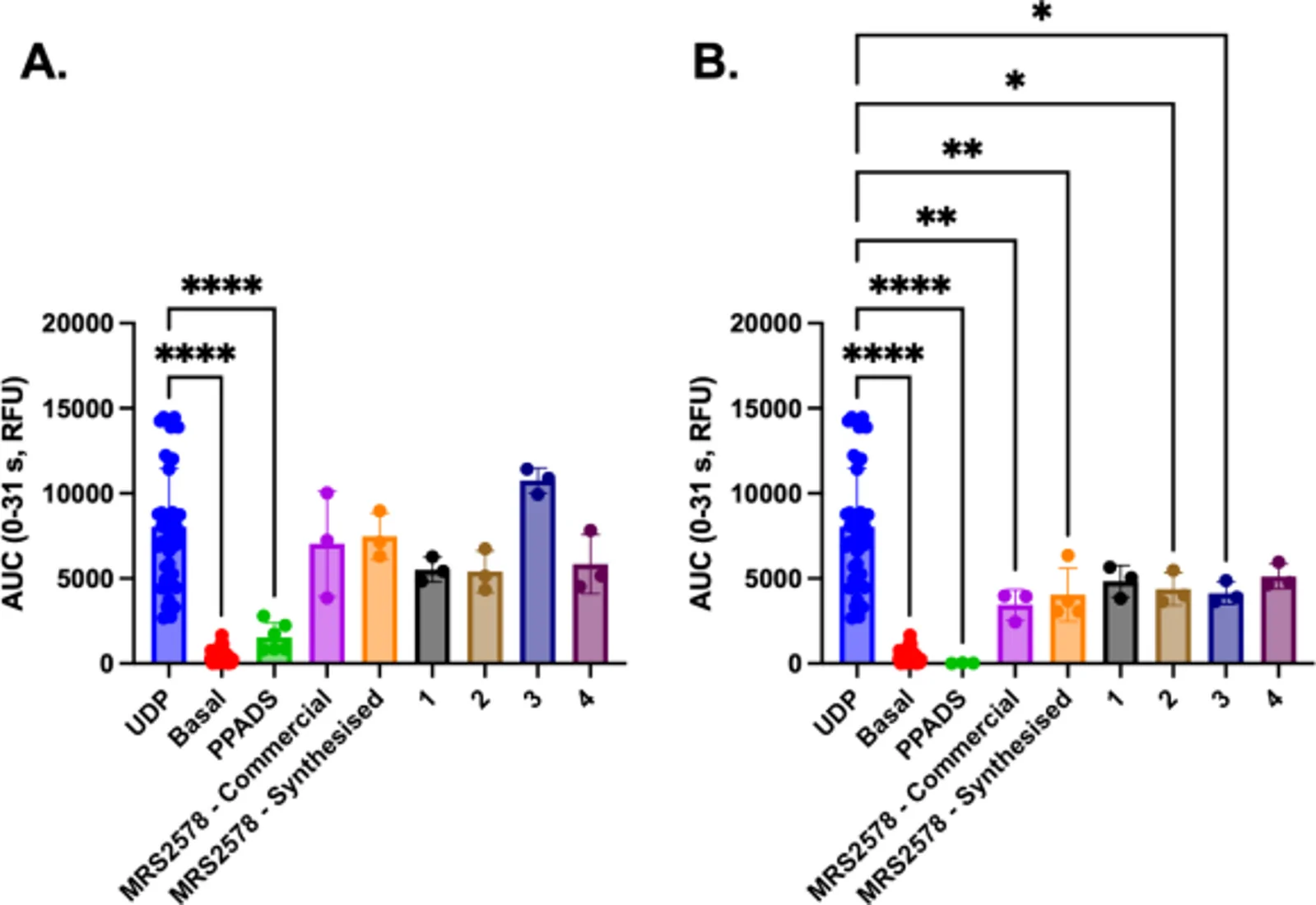
Fluorescent values generated by downstream Ca^2+^ following treatment of 132-1N1 cells stably expressing the hP2Y6R with an EC_80_ concentration of UDP (75 nM). Potential antagonists were screened 100 µM (PPADS) or 10 µM (MRS2578, 1–4) and either (**A**) not pre-treated or (**B**) pre-treated for 30 min. Experiments were performed in duplicate, and the data is the mean ± SD (*n* ≥ 3 experiments, ***p < 0.001, ****p < 0.0001)

The functional activity of compounds **1–4** were evaluated using 132-1N1 cells transfected with the hP2Y6R for antagonism (Fig. 6). Utilising downstream G_q_ pathways of the GPCR P2Y6R, the antagonistic potential of compounds **1–4** were assessed with non-selective P2 antagonist pyridoxal phosphate-6-azophenyl-2’,4’-disulfonic acid tetrasodium salt (PPADS) and the selective commercially-available P2Y6R antagonist MRS2578 by their ability to inhibit UDP-induced Ca^2+^ mobilisation.

Challenging the P2Y6R with an EC_80_ concentration of UDP (75 nM) determined using an agonist assay with the same cells (Supplementary Table 3), we first exposed the cells to both agonist and antagonist at the same time without pre-incubation. Compounds **1–4** and MRS2578 were initially screened at 10 µM, over 10-fold higher than their reported IC_50_’s. In contrast, PPADS was screened at 100 µM. The higher concentration was justified as previous reports have shown PPADS to only reduce UDP-mediated P2Y6R signalling by 31% at 100 µM [40]. Under the experimental conditions of this study, compounds **1–4** and both commercial and synthesised (Supplementary 4) sources of MRS2578 demonstrated no antagonistic activity at the P2Y6R despite reports in the literature, while PPADS significantly reduced P2Y6R mediated Ca^2+^ flux (AUC = 1540 ± 849, *p* < 0.0001, Fig. 6A). These results were similarly reported by Jacobson and coworkers in their latest publication (Oliva et al., 2024), declaring compound **1** to only be a weak hP2Y6R antagonist (18.9 ± 8.5% inhibition at 100 µM) [22]. Jacobson and coworkers did not screen any of the other literature compounds discussed herein. Moreover, it is worth noting that although MRS2578 has been used in various studies as a potent and selective P2Y6R antagonist, the inhibitory effects have been demonstrated to vary in different cells or tissues [17]. This variance has been suggested to be due to its limited stability and solubility in aqueous solution, which has been supported in literature extensively [11, 18, 19]. In our experimental conditions MRS2578, though dissolved initially in DMSO, precipitated out of solution when added to the experimental buffer solution. Attempts to improve solubility were conducted including warming MRS2578 to 37 °C prior to adding to HBSS buffer, pre-diluting in HBSS (1:4) as well as using different commercial sources of MRS2578 (MedChemExpress and Sigma-Aldrich) and synthesising MRS2578 in-house (Supplementary 4). The buffer solutions from Oliva et al. (2024), Zhao et al. (2024) and Zhu et al. (2023) were also replicated to no prevail [22–24]. This could explain why no antagonism was observed.

It was postulated that the discrepancy observed between our results and those reported by the original research papers could be rationalised by the differences between in vitro assay methodology (Supplementary 3). Four key assay parameters were varied in this work compared to the original papers: antagonist pre-incubation, agonist concentration, agonist exposure time and cell-type. The Zhu et al. (2023) paper and Oliva et al. (2024) papers included a pre-treatment of cells with compounds for 30 min and 45 min, respectively, prior to addition of UDP, whereas Zhao et al. (2024) failed to disclose whether their methodology including a pre-treatment step [22–24]. Therefore, the Ca^2+^ flux assay was repeated as stated previously with the inclusion of a 30 min pre-incubation step (Fig. 6B). Interestingly, PPADS (AUC = 26.89 ± 24.57 *p* < 0.001) significantly reduced UDP-P2Y6R-mediated Ca^2+^ flux to levels similar to that seen in basal conditions (AUC = 475.5 ± 899.5). This observed antagonism may suggest the slow kinetics of PPADS required to bind with the P2Y6R to elicit an effect. Within our system, both commercial and synthesised MRS2578 produced a reduction in Ca^2+^ flux levels but not to zero. This was attributed to the significant solubility issues we encountered when adding MRS2578 into our aqueous buffer solution, a frequently reported shortcoming of the compound [11, 18, 19]. Further, Zhu et al. (2023) or Zhao et al. (2024) reported compounds (**1–4**) elicited a small reduction in fluorescent signal when pre-incubated at 10 µM. Compound **1** reduced UDP signalling to 66.5 ± 12.78 which is in agreement with that seen by Jacobson and colleagues [22]. Compounds **2**, **3** and **4** similarly only reduced UDP-mediated calcium fluorescence to 60.0 ± 13.3%, 56.8 ± 8.96% and 70.3 ± 9.98%, respectively. Though a slight reduction, these compounds in our assay do not recapitulate the total inhibition of UDP-P2Y6R activity at 10 µM seen in the original articles by Zhu et al. (2023) and Zhao et al. (2024).

**Fig. 7:**
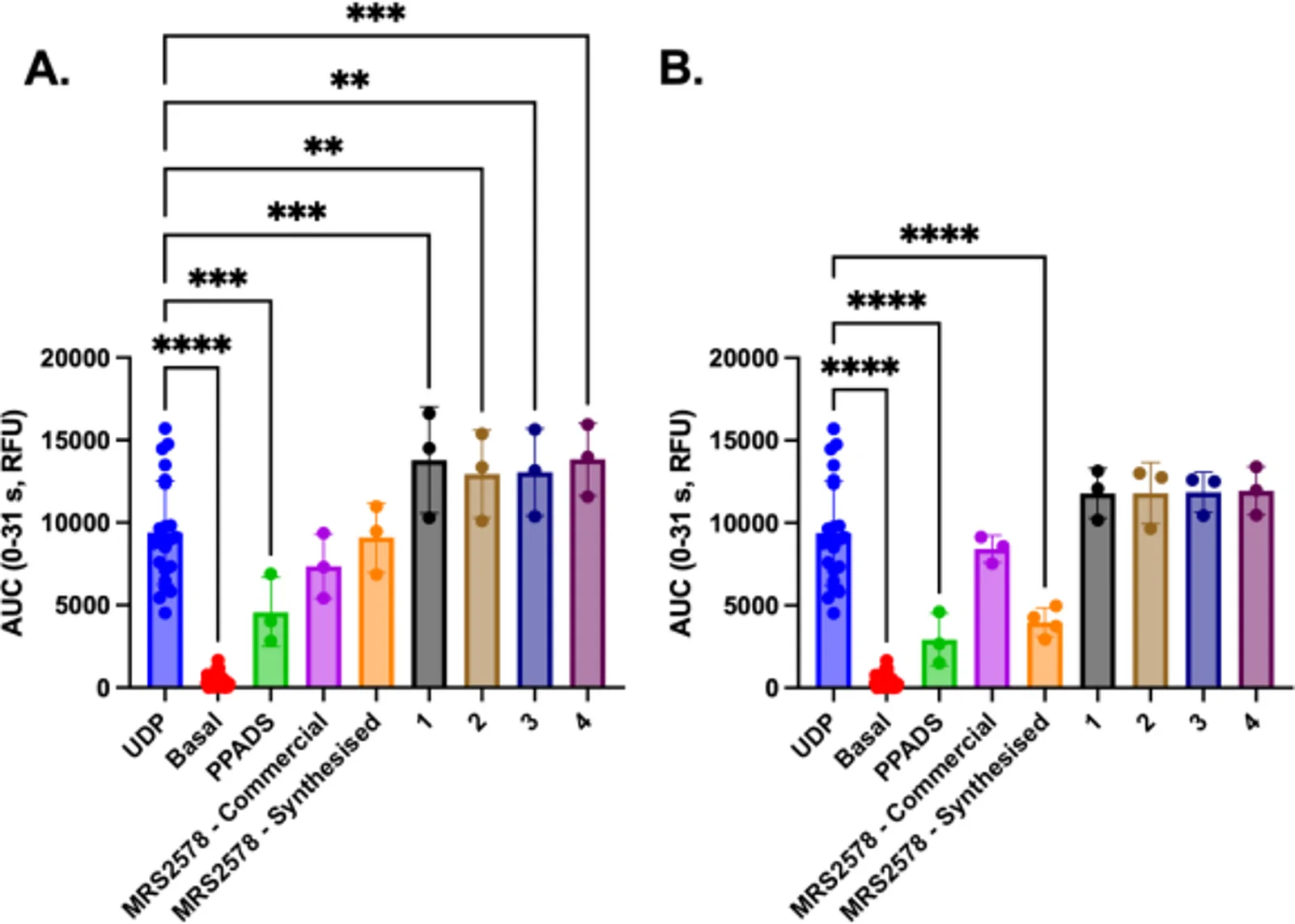
Fluorescent values generated by downstream Ca^2+^ following treatment of 132-1N1 cells stably expressing the hP2Y6R with a saturating concentration of UDP (10,000 nM). Potential antagonists were screened 100 µM (PPADS) or 10 µM (MRS2578, **1–4**) and either (**A**) not pre-treated or (**B**) pre-treated for 30 min. Experiments were performed in duplicate, and the data is the mean ± SD (*n* ≥ 3 experiments, ***p < 0.001, ****p < 0.0001)

Zhu et al. (2023) utilised a UDP concentration of 10,000 nM, which is well over the reported EC_50_ (750 nM), while Zhao et al. (2024) did not disclose the UDP level used [23, 24]. We therefore repeated the experiments above, either with or without antagonist pre-treatment with modification of 10,000 nM of UDP (Fig. 7A, B). We observed a similar trend in results to that seen against 75 nM UDP. No compounds were able to antagonise P2Y6R-mediated Ca^2+^ mobilisation with the exception of PPADS and synthesised MRS2578. At 100 µM PPADS significantly reduced the generated fluorescent signal by 10,000 nM UDP (AUC = 2920 ± 1570, *p* < 0.0001), when pre-treated for 30 min. This was seen to a lesser extent in the absence of pre-treatment (AUC = 4583 ± 2095, *p* < 0.001 Fig. 7A). Synthesised MRS2578 reduced Ca^2+^ mobilisation only when pre-treated prior to UDP addition, reducing UDP-mediated activity to 47.8 ± 18.2%. Compounds **1–4** without pre-incubation appear to significantly increase Ca^2+^ mobilisation in no pre-treatment conditions with a greater AUC. Therefore, these results indicate that the UDP concentration employed may influence the presence or absence of antagonistic activity of **1–4**.

**Fig. 8:**
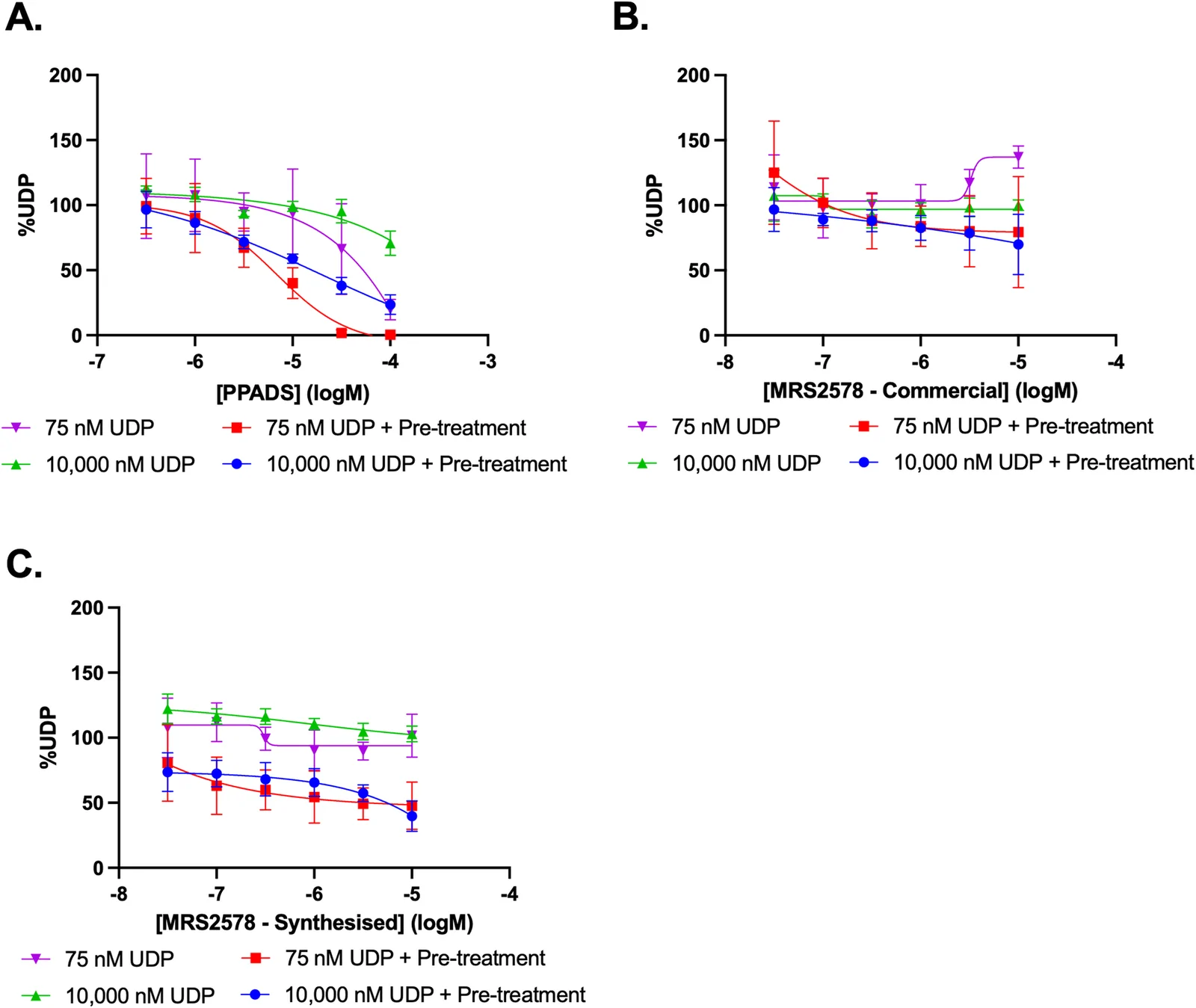
Concentration-response curve of PPADS (**A**), MRS2578 Commercial (**B**) and Synthesised (**C**) on hP2Y6R expressing 132-1N1 cells against UDP (75 nM or 10,000 nM) with or without pre-treatment (30 min). Data is the mean ± SD (*n* ≥ 3 experiments)

In light of only PPADS and MRS2578 commercial and synthesised sources, depending on conditions, producing an antagonistic effect on 132-1N1-hP2Y6R cells, we further conducted a concentration-response experiment in similar conditions as outlined previously. PPADS was only able to generate an IC_50_ of 7962 ± 1548 nM when challenged with 75 nM UDP following pre-treatment (Fig. 8). Omitting a pre-incubation step, resulted in a loss of antagonism, only reducing UDP-induced Ca^2+^ flux to 19.82 ± 7.91% at 100 µM, aligning with the reported 31% reduction of P2Y6R activity at the same concentration in literature [40]. Challenging with 10,000 nM saw a reduction in antagonism (Fig. 8). Despite a concentration-response curve fit, this level of UDP was insurmountable by PPADS, particularly in no-pre-treatment conditions with a reduction to 70.80 ± 13.27% UDP. At such high levels of UDP, it is rational to hypothesise that UDP has off-target interactions leading to an independent pathway increasing Ca^2+^ that is not antagonised by PPADS. One potential pathway is *via* the P2Y14R, which has no reported evidence of PPADS antagonism despite UDP acting as an agonist [41]. Commercial MRS2578 was unable to produce a concentration-response curve in any conditions, which can be attributed to the severity of precipitation observed when added to experimental aqueous buffer solutions (Fig. 8B). The synthesised MRS2578 reduced UDP-induced Ca^2+^ flux in both conditions where the compound was pre-treated for 30 min to approximately 40% at 10 µM and a concentration dependent return to 100% as MRS2578 concentrations decreased (Fig. 8C). However, synthesised MRS2578 could not completely abolish UDP-mediated Ca^2+^ flux which can be explained by the precipitation of the compound out of solution, potentially reducing the concentration of soluble compound available to bind with the P2Y6R.

**Fig. 9:**
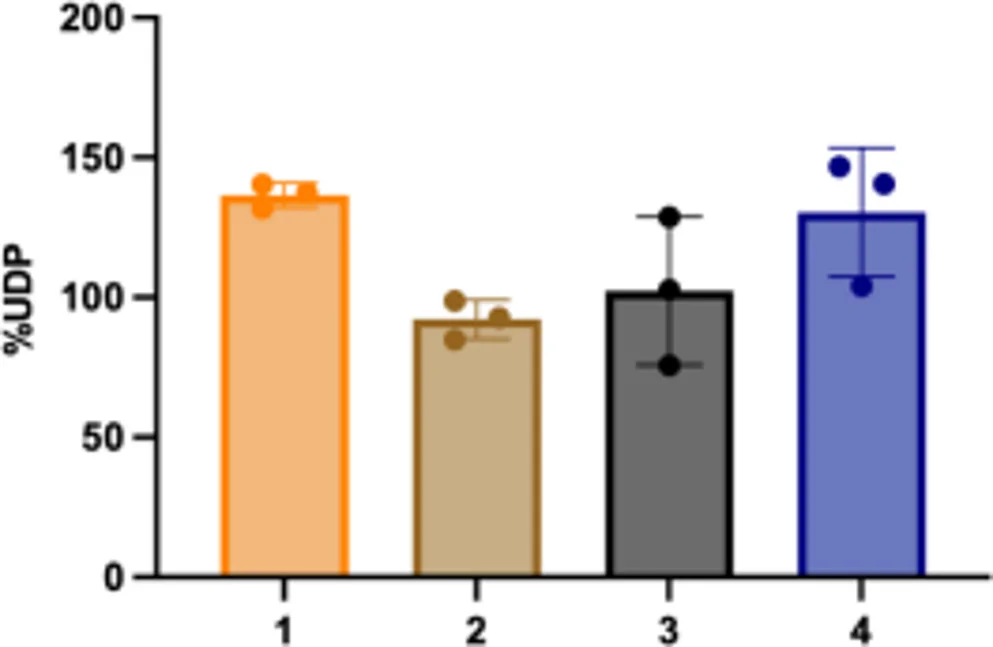
Percentage inhibition of control agonist (UDP) response. Inhibition was evaluated in a Ca^2+^ flux assay following treatment of 132-1N1 cells stably expressing the hP2Y6R with UDP (10 nM). Experiments were performed by Eurofins Scientific in triplicate, and the data is the mean ± SD (*n* ≥ 3 experiments)

Given the discrepancies with previously reported findings, further evaluation was conducted by an independent contract research organisation (Eurofins Scientific) to assess compounds **1–4**. Consistent with our results, compounds **1–4** were found to have no inhibition of UDP-stimulated Ca^2+^ levels in their P2Y6R antagonist Ca^2+^ flux assay (Fig. 9).

**Fig. 10:**
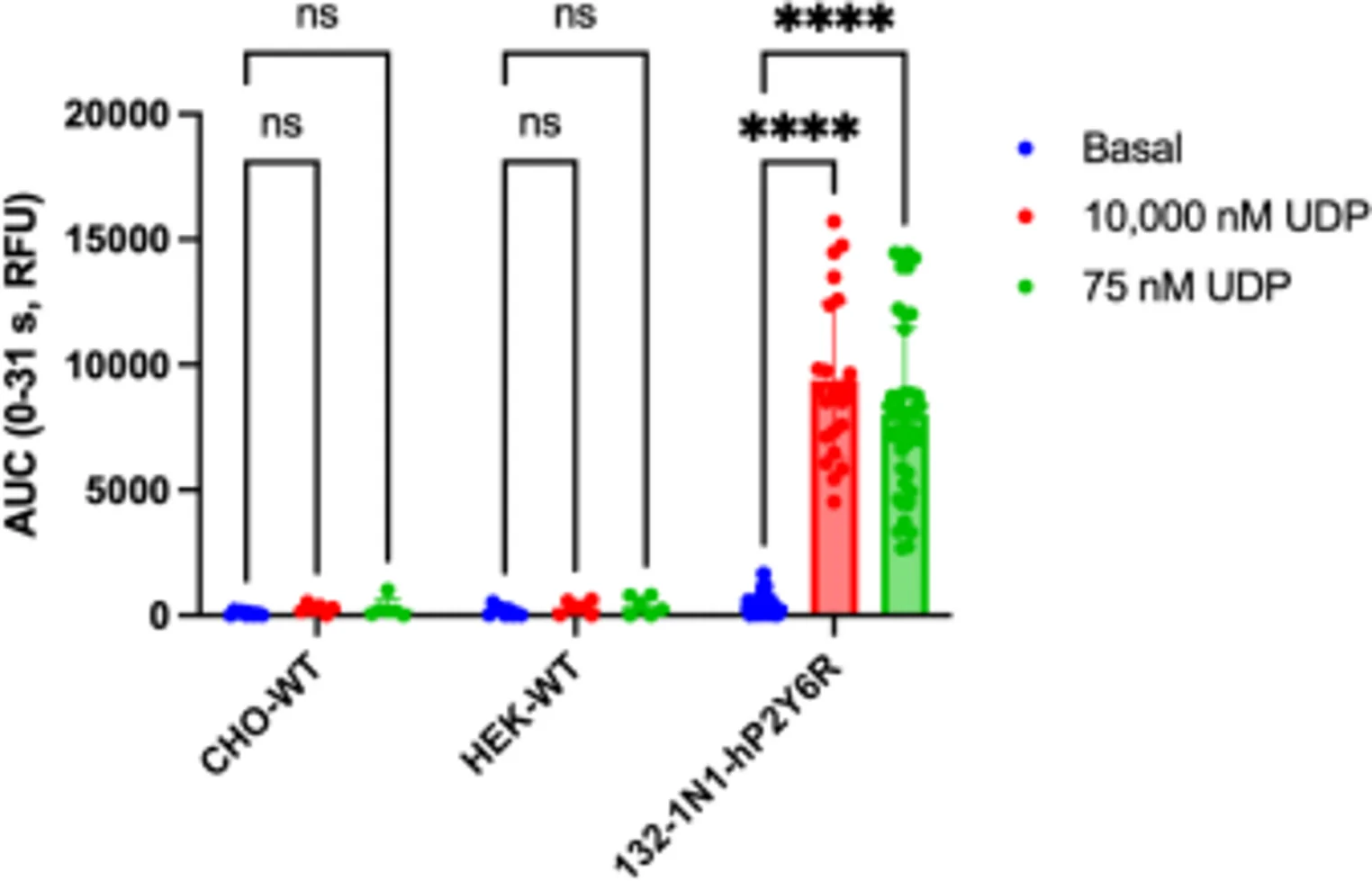
Fluorescent values generated by downstream Ca^2+^ following treatment of CHO, HEK and/or 132-1N1 cells stably expressing the hP2Y6R with an EC_50_ (75 nM) or saturating concentration of UDP (10,000 nM). Experiments were performed in duplicate, and the data is the mean ± SD (*n* ≥ 3 experiments, ****p < 0.0001, ns = non-significant)

With compound **1–4** unable to achieve antagonism of the P2Y6R in multiple Ca^2+^ mobilisation assays, further investigation of the methodologies utilised were explored (Supplementary 3). Of the differences, cell type may have contributed to the disjunction in results between studies. The cell types used by those that observed antagonism included HEK293 (Li et al., 2022; Zhu et al., 2023) and Chinese hamster ovary (CHO, Zhao et al., 2024) cells stably expressing the human P2Y6R, while the three that did not detect antagonism all used 132-1N1 cells stably expressing the human P2Y6R (This work, Eurofins Scientific and Oliva et al., 2024) [22–25]. As the patent disclosure by Li et al. (2022) and publication Zhu et al. (2023) belong to the same research group, the methodology of screening is the same [23, 25]. Stably-expressing cell lines are generally used for their ease of transfection and scalability and are considered “blank slates”. However, without genetic intervention they all possess basal expression of GPCRs to differing levels. Woszczek et al. (2011) reported low levels of P2Y6R mRNA in untransfected HEK293 cells [42]. Yet, the same cells observed Ca^2+^ flux in response to 100 µM UDP that could not be antagonised by MRS2578, suggesting a potentially different pathway of UDP-mediated Ca^2+^-flux [42]. A broad approach study identified mRNA expression of over 75 different GPCRs on untransfected HEK293 cells, including P2Y1R and P2Y2R. Additionally functional assays confirmed increased Ca^2+^ signalling *via* endogenously expressed P2Y1R and P2Y2R on HEK293 cells transfected with CXR2 [43]. Separately, mRNA expression of P2Y11R was found on HEK293 cells [44]. Similarly, P2YR-associated increase in Ca^2+^ signalling was detected on untransfected CHO cells following stimulation with 2MeSATP and ATP [45]. ATP is reported as an agonist of the P2Y2R and P2Y11R, while 2MeSATP is a partial agonist to P2Y12R [46, 47]. Conversely, native 132-1N1 cells do not respond to endogenous nucleotides and are subsequently absent of endogenous P2R activity [48, 49]. Further Zhu et al. (2023) used considerably high levels of UDP (10,000 nM), as stated previously is over 10-fold higher than their reported EC_50_ [23]. With the exception of Zhao et al. (2024) who did not disclose UDP concentration data, the rest of the research used much lower levels (≤ 100 nM) [24]. At such high concentrations UDP may be causing off target Ca^2+^ signalling which could potentially be blocked by the novel antagonists in a manner not seen at the lower concentrations. An example is the P2Y14R, where UDP has been evidenced as a potent agonist in hP2Y14R stably expressing HEK293, CHO and C6 cells with EC_50_ values of 74, 33 and 29 nM, respectively, in Ca^2+^ flux assays [41]. To confirm off-target UDP mediated Ca^2+^ flux, we challenged HEK and CHO wildtype cells against 75 UDP and 10,000 nM UDP. We found that there was no significant increase in Ca^2+^ flux in response to either concentration of UDP in each cell type, while there was a significant increase in 132-1N1 astrocytoma cells stably expressing the human P2Y6R (Fig. 10). Despite no UDP activity in wild type cells, it is possible that **1–4** are antagonists to another pathway and highlights the notion that choosing a suitable cell line and agonist condition is paramount to ensuring the results obtained are directly caused by the receptor of interest.

Under the experimental conditions of this study, compounds **1–4** are not considered as antagonists of the P2Y6R. However, this study has potential limitations with activity only assessed through a calcium mobilisation assay [50]. A more robust evaluation of the functional activity of compounds **1–4** would benefit from conducting the Ca^2+^ flux assay herein using the same P2Y6R expressing cells utilised in Zhu et al. (2023) and Zhao et al. (2024).

Additionally, supplementary radioligand binding assays to confirm target engagement and selectivity profiling amongst P2 receptor subtypes would provide valuable insight into the translatability of these chemotypes.

## Conclusions

The results from this work demonstrate that the previously reported compounds (**1–4**) exhibit no notable activity at the hP2Y6R when assessed under the experimental conditions of this study. These results were consistently observed in 132-1N1 cells, both in the presence and absence of a pre-incubation step with varying concentrations of agonist, UDP. Importantly, these observations were supported by a third-party research organisation and aligned with additional reported literature [22]. In silico molecular modelling experiments similarly produced conflicting results to those contained in the original published literature, with both leads **1** and **2** failing redocking experiments [23, 24]. Collectively, these results highlight the need for continued efforts in establishing a more accurate homology model of the P2Y6R to better support future structure-based drug design. The current work serves as a cautionary example of the methodological inconsistencies that can arise when assessing P2Y6R functional activity, therefore highlighting the urgent need for robust, reproducible functional assays – a critical hurdle that must be overcome to allow meaningful translation of P2Y6R antagonists toward clinical development.

## Materials and Methods/Experimental

### Synthetic Experimental

#### *General Information for Chemical Synthesis*

Reagents were obtained from commercial sources and used without further purification unless otherwise stated. Dry solvents were obtained from a PureSolv MD 7 solvent purification system (Innovative Technology, Inc.). Reactions were conducted under a positive pressure of a dry nitrogen atmosphere unless otherwise specified. Analytical thin-layer chromatography (TLC) were performed on Merck 0.2 mm aluminium-backed silica gel 60 F254 plates which were visualised with shortwave (254 nm) and/or longwave (365 nm) ultraviolet (UV) light. Compounds without a UV active chromophore were visualised with *p*-anisaldehyde, cerium molybdate, potassium permanganate or vanillin. Flash chromatography was performed using Merck silica gel 60 (230–430 mesh). All solvent compositions are reported as their volume/volume ratios. Infrared (IR) absorption spectra were recorded on a Bruker ALPHA FT-IR spectrometer, with the data reported as vibrational frequency (cm^− 1^). Nuclear magnetic resonance (NMR) spectra were recorded on either a Bruker AVANCE DRX300 (300 MHz), DRX400 (400 MHz) or AVANCE II 500 Ascend (500 MHz) spectrometer at 300 K. Chemical shifts (*δ*, ppm) are reported relative to the residual solvent peak. Multiplicities are assigned as singlet (s), doublet (d), triplet (t), quartet (q), pentet (p), doublet of doublets (dd), or multiplet (m), with the observed coupling constant (*J*, Hz) described in Hertz. Nuclear assignments were performed with the aid of COSY, NOSEY, HMBC and HSQC experiments where necessary. Low-resolution mass spectrometry (LRMS) was performed using a Bruker© amaZon Speed-Ion Trap mass spectrometer using electrospray ionisation (ESI) or atmospheric pressure chemical ionization (APCI). Samples run by ESI were injected (20 µL) into a continuous flow of methanol or acetonitrile (0.3 mL/min) with an Alltech HPLS pump, and a direct probe injection method was utilized for APCI (corona current 4000 nA, vaporization temperature 400 °C). High resolution mass spectrometry (HRMS) was recorded on a Bruker 7T Apex Qe Fourier Transform Ion Cyclotron resonance mass spectrometer equipped with an Apollo II ESI/APCI/MALDI Dual source. Purity analysis was performed by analytical highperformance liquid chromatography (HPLC) on a Waters© Alliance 2695 apparatus equipped with Waters 2996 photodiode array detector, set at 254 nm. Separation using a SunFireTM C18 column (5 μm, 2.1 × 150 mm) was achieved using water (solvent A) and acetonitrile (solvent B) at a flow rate of 0.2 mL/min. The method consisted of 0% B to 100% B over 30 min. HPLC data is recorded as percentage purity and retention time (R_T_) in minutes.

#### ethyl 4-(furan-2-yl)-2,4-dioxobutanoate (**7**)

A suspension of *t*-BuOK (3.76 g, 33.0 mmol) in anhydrous THF (10 mL) was cooled to 0 °C. Commercially available 2-acetylfuran (1.88 g, 17.0 mmol) and diethyl oxalate (4.4 mL, 32.0 mmol) in 1,2-dimethoxyethane (10 mL) was added dropwise to the stirring suspension. The reaction was allowed to come to room temperature over the next hour, and a precipitate formed. Upon consumption of acetyl furan, confirmed *via* TLC, the mixture was quenched with 1 M solution of HCl (17 mL, 17.0 mmol), dissolving the precipitate. The crude product was extracted with EtOAc (3 × 25 mL/mmol) and the pooled organic phase was washed with brine (1 × 25 mL/mmol) and dried over MgSO_4_. Compound **157** was obtained as yellow crystals (2.98 g, 83%) following purification by flash chromatography (1:49 EtOAc: Hex). ^**1**^**H NMR** (400 MHz, CDCl_3_) *δ* 14.45 (s, 1 H), 7.67 (dd, *J* = 1.7, 0.8 Hz, 1 H), 7.34 (dd, *J* = 3.7, 0.8 Hz, 1 H), 6.93 (s, 1 H), 6.61 (dd, *J* = 3.6, 1.7 Hz, 1 H), 4.38 (q, *J* = 7.2 Hz, 2 H), 1.40 (t, *J* = 7.1 Hz, 3 H) ppm; ^**13**^**C NMR** (101 MHz, CDCl_3_) *δ* 181.1, 166.3, 162.1, 151.1, 147.8, 118.5, 113.2, 99.1, 62.7, 14.2 ppm; **LRMS (+ ESI)** m/z: 233 ([M + Na]^+^, 100), **(-ESI)** m/z: 209 ([M-H]^−^, 100); **HRMS (+ ESI)** Calc. for C_10_H_10_NaO_5_ [M + Na]^+^: 233.04204, found 233.04207. Matched previously reported characterisation data [51].

#### ethyl 1-(*tert*-butyl)-5-(furan-2-yl)-1 H-pyrazole-3-carboxylate (**8**)

A solution of **7** (1.52 g, 7.23 mmol) in EtOH (15 mL) was treated with *tert*-butylhydrazine hydrochloride (1.24 g, 9.95 mmol) at room temperature and stirred for 8 h. The solvent was evaporated and the residue taken up in EtOAc (1 × 25 mL/mmol), washed with H_2_O (1 × 25 mL/mmol), brine (1 × 25 mL/mmol), dried over MgSO_4_ and concentrated *in vacuo*. Compound **8** was obtained as a yellow oil (1.78 g, 94%) following purification by flash chromatography (1:4 EtOAc: Hex). ^**1**^**H NMR** (500 MHz, CDCl_3_) *δ* 7.55 (dd, *J* = 1.7, 0.7 Hz, 1 H), 6.87 (s, 1 H), 6.49 (dd, *J* = 3.3, 1.8 Hz, 1 H), 6.47 (dd, *J* = 3.3, 0.7 Hz, 1 H), 4.38 (q, *J* = 7.1 Hz, 2 H), 1.55 (s, 9 H), 1.38 (t, *J* = 7.1 Hz, 3 H) ppm; ^**13**^**C NMR** (126 MHz, CDCl_3_) *δ* 162.6, 143.9, 143.0, 140.7, 133.5, 113.9, 112.2, 111.5, 62.7, 60.9, 30.0, 14.5 ppm; **LRMS (+ APCI)** m/z: 263 ([M + H]^+^, 100); **LRMS (+ ESI)** m/z: 285 ([M + Na]^+^, 100); **HRMS (+ ESI)** Calc. for C_14_H_19_N_2_O_3_ [M + H]^+^: 263.13902, found 263.13932. Matched previously reported characterisation data [51].

#### (1-(*tert*-butyl)-5-(furan-2-yl)-1 H-pyrazol-3-yl) methanol (**9**)

A solution of **8** (1 g, 3.81 mmol) in THF (5 mL) was treated dropwise with LiAlH_4_ (1 M in THF, 6.8 mL, 6.81 mmol) at 0 °C. The reaction mixture was then stirred at 0 °C for 30 min and allowed to warm to rt over 1 h, followed by heating to reflux for 2 h. Following complete conversion of starting material by TLC, the reaction was cooled to 0 °C and quenched by sequential addition of H_2_O (1 mL/g LiAlH_4_), 4 M aq. NaOH (1 mL/g LiAlH_4_) and H_2_O (3 mL/g LiAlH_4_). The crude mixture was filtered through a pad of celite^®^ and washed with EtOAc (3 mL). Combined filtrates were concentrated *in vacuo* to yield **9** as a white solid (749 mg, 89%). ^**1**^**H NMR** (400 MHz, CDCl_3_) *δ* 7.52 (dd, *J* = 1.9, 0.9 Hz, 1 H), 6.47 (dd, *J* = 3.3, 1.9 Hz, 1 H), 6.42 (dd, *J* = 3.3, 0.9 Hz, 1 H), 6.34 (s, 1 H), 4.67 (s, 2 H), 1.49 (s, 9 H) ppm, O*H* signal not observed; ^**13**^**C NMR** (101 MHz, CDCl_3_) *δ* 148.9, 144.9, 142.6, 133.1, 111.6, 111.3, 109.3, 61.1, 59.2, 30.2 ppm; **LRMS (+ APCI)** m/z: 221 ([M + H]^+^, 100); **HRMS (+ APCI)** Calc. for C_12_H_17_N_2_O_2_ [M + H]^+^: 221.12845, found 221.12847.

#### 1-(*tert*-butyl)-5-(furan-2-yl)-1 H-pyrazole-3-carbaldehyde (**5**)

DMP (1.85 g, 4.36 mmol) was added portion wise to a solution of alcohol **9** (570 mg, 2.6 mmol) in CH_2_Cl_2_ (15 mL) at 0 °C. The resultant slurry was stirred for 1 h at rt. Upon completion, the reaction contents were quenched with sat. aqueous Na_2_S_2_O_3_ (5 mL) and sat. aqueous NaHCO_3_ (5 mL). The resultant biphasic system was stirred vigorously for 5 min at rt, then filtered over a pad of celite. The filtrate was extracted with CH_2_Cl_2_, washed with brine, dried and concentrated *in vacuo*. Compound **5** was isolated as a white solid (530 mg, 94%) following purification by flash chromatography (1:9 EtOAc: Hex). ^**1**^**H NMR** (400 MHz, CDCl_3_) *δ* 9.96 (s, 1 H), 7.56 (dd, *J* = 1.8, 0.9 Hz, 1 H), 6.88 (s, 1 H), 6.52–6.48 (m, 2 H), 1.56 (s, 9 H) ppm; ^**13**^**C NMR** (101 MHz, CDCl_3_) *δ* 186.9, 148.5, 143.6, 143.2, 134.4, 112.5, 111.6, 110.7, 63.0, 30.0 ppm; **LRMS (+ APCI)** m/z: 219 ([M + H]^+^, 100) ppm; **HRMS (+ APCI)** Calc. for C_12_H_15_N_2_O_2_ [M + H]^+^: 219.11280, found 219.11288.

#### 2-(1-(*tert*-butyl)-5-(furan-2-yl)-1 H-pyrazol-3-yl)-5-chloro-1Hbenzo[d]imidazole (*5(6)*-**1**)

Compound **5** (40.2 mg, 184 µmol), 4-chlorobenzene-1,2-diamine (31.4 mg, 220 µmol) and sodium bisulfite (40.5 mg, 389 µmol) were dissolved in a solution of EtOH/H_2_O (2:1, 2 mL) and refluxed for 1 h. The reaction solution was extracted with EtOAc (3 × 25 mL/mmol), dried with MgSO_4_, concentrate *in vacuo*. Compound **1** was isolated as a white solid (47 mg, 75%) following purification by flash chromatography (1:4 EtOAc: Hex). Characterisation data is reported as a complex mix of tautomers. ^**1**^**H NMR** (500 MHz, DMSO-*d*_*6*_) *δ* 12.94–12.70 (m, 1 H), 7.91 (d, *J* = 1.7 Hz, 1 H), 7.70–7.59 (m, 1 H), 7.54–7.49 (m, 1 H), 7.26–7.16 (m, 1 H), 7.03 (s, 1 H), 6.76 (d, *J* = 3.3 Hz, 1 H), 6.67 (dd, *J* = 3.3, 1.9 Hz, 1 H), 1.55 (s, 9 H) ppm; ^**13**^**C NMR** (126 MHz, DMSO-*d*_*6*_) *δ* 147.9, 147.5, 144.6, 143.9, 143.0, 142.5, 139.8, 135.2, 133.5, 133.3, 126.7, 125.9, 122.6, 121.9, 119.9, 118.1, 112.8, 112.5, 111.8, 111.2, 110.1, 61.8, 29.6 ppm; 2D Spectra available in Appendix 6; **LRMS (+ ESI)** m/z: 341/343 ([M + H]^+^, 100), 363/365 ([M + Na]^+^, 100), **(-ESI)** m/z: 339/341 ([M-H]^-^, 100); **HRMS (+ ESI)** Calc. for C_18_H_18_ClN_4_O [M + H]^+^: 341.11637/343.11342, found 341.11696/343.11421; **HPLC** R_T_ = 21.04 min, 99.70% (254 nm).

#### 2-(1-(*tert*-butyl)-5-(furan-2-yl)-1 H-pyrazol-3-yl)-1 h-benzo[d]-imidazole (**3**)

Compound **5** (29.1 mg, 133 µmol), *o*-phenyldiamine (23.4 mg, 216 µmol) and sodium bisulfite (55.5 mg, 533 µmol) were dissolved in a solution of EtOH/H_2_O (2:1, 2 mL) and refluxed for 1 h. The reaction solution was extracted with EtOAc (3 × 25 mL/mmol), dried with MgSO_4_, and concentrated *in vacuo*. Compound **3** was isolated as a white solid (26.5 mg, 65%) following purification by flash chromatography (1:4 EtOAc: Hex). ^**1**^**H NMR** (400 MHz, CDCl_3_) *δ* 9.96 (s, 1 H), 7.79 (s, 1 H), 7.57 (t, *J* = 1.4 Hz, 1 H), 7.46 (s, 1 H), 7.27 (s, 1 H), 7.25 (d, *J* = 3.2 Hz, 1 H), 6.51 (d, *J* = 1.2 Hz, 2 H), 1.59 (s, 9 H) ppm; ^**13**^**C NMR** (101 MHz, CDCl_3_) *δ* 147.2, 144.1, 142.9, 140.2, 134.2, 123.3, 122.3, 119.8, 112.2, 111.6, 110.7, 110.2, 62.1, 30.1 ppm; **LRMS (+ ESI)** m/z: 307 ([M + H]^+^, 100), **(-ESI)** m/z: 305 ([M-H]^-^, 100); **HRMS (+ ESI)** Calc. for C_18_H_19_N_4_O [M + H]^+^: 307.15534, found 307.15540; **HPLC** R_T_ = 19.11 min, 99.5% (254 nm).

#### 1-(1*H*-pyrrol-2-yl)-3-(quinolin-3-yl)prop-2-en-1-one (**12**)

To a solution of quinoline-3-carbaldehyde (200 mg, 1.27 mmol) and 2-acetylpyrrole (142 mg, 1.30 mmol) in EtOH (2 mL) was added aq. NaOH (2.5 M, 1 mL, 2.54 mmol). The reaction was stirred at rt for 5 h, after which the resulting precipitate was filtered and washed with EtOH. Compound **12** was obtained as a pale yellow powder (218 mg, 69%) following filtration. ^**1**^**H NMR** (500 MHz, DMSO-*d*_*6*_) *δ* 12.05 (s, 1 H), 9.41 (d, *J* = 2.2 Hz, 1 H), 8.78 (d, *J* = 2.1 Hz, 1 H), 8.06 (dd, *J* = 8.4, 1.1 Hz, 1 H), 8.01 (dd, *J* = 8.2, 1.4 Hz, 1 H), 8.00 (d, *J* = 15.7 Hz, 1 H), 7.84 (d, *J* = 15.7 Hz, 1 H), 7.82–7.79 (m, 1 H), 7.67 (ddd, *J* = 8.1, 6.8, 1.2 Hz, 1 H), 7.46 (dd, *J* = 3.8, 1.4 Hz, 1 H), 7.21 (dd, *J* = 2.4, 1.4 Hz, 1 H), 6.31 (dd, *J* = 3.8, 2.4 Hz, 1 H) ppm; ^**13**^**C NMR** (126 MHz, DMSO-*d*_*6*_) *δ* 177.4, 150.2, 147.8, 137.4, 135.6, 133.0, 130.4, 128.9, 128.6, 128.2, 127.5, 127.3, 126.8, 124.9, 117.9, 110.3 ppm; **LRMS (-ESI)** m/z: 247 ([M-H]^−^, 100); **HRMS (+ ESI)** Calc. for C_16_H_12_N_2_NaO [M + Na]^+^: 271.08418, found 271.08371.

#### 4-(3-(1*H*-pyrrol-2-yl)-5-(quinolin-3-yl)-4,5-dihydro-1*H*-pyrazol-1-yl)-4-oxobutanoic acid (**2**)

To a solution of **12** (50.3 mg, 0.20 mmol) in EtOH (1 mL) was added hydrazine monohydrate (50 µL, 1.01 mmol). The reaction was stirred at 80 °C for 2 h. Following consumption of starting material as indicated by TLC, the reaction was added to ice H_2_O. The resulting precipitate was filtered, washed with EtOH and dried to obtain the dihydropyrazole analogue **14**. The residue was dissolved in CHCl_3_ (2 mL) and treated with succinic anhydride (39.2 mg, 0.39 mmol). The reaction was stirred at reflux 4 h. The precipitate was filtered and washed with EtOAc to yield compound **2** as a grey solid (30.3 mg, 41% over two steps). ^**1**^**H NMR** (500 MHz, DMSO-*d*_*6*_) *δ* 12.09 (s, 1 H), 11.58 (s, 1 H), 8.81 (d, *J* = 2.2 Hz, 1 H), 8.08 (d, *J* = 2.4 Hz, 1 H), 8.00 (d, *J* = 8.5 Hz, 1 H), 7.95 (d, *J* = 8.2 Hz, 1 H), 7.73 (t, *J* = 7.7 Hz, 1 H), 7.59 (t, *J* = 7.5 Hz, 1 H), 6.98 (d, *J* = 3.3 Hz, 1 H), 6.56–6.51 (m, 1 H), 6.15 (q, *J* = 2.8 Hz, 1 H), 5.72 (dd, *J* = 11.7, 4.7 Hz, 1 H), 3.86 (dd, *J* = 17.8, 11.8 Hz, 1 H), 3.22 (dd, *J* = 17.8, 4.7 Hz, 1 H), 3.09 (dt, *J* = 16.9, 6.9 Hz, 1 H), 2.91 (dt, *J* = 17.0, 6.6 Hz, 1 H), 2.58–2.43 (m, 2 H) (overlapping with solvent peak) ppm; ^**13**^**C NMR** (126 MHz, DMSO-*d*_*6*_) *δ* 173.9, 168.6, 149.5, 148.2, 146.9, 135.0, 131.9, 129.3, 128.6, 128.1, 127.4, 126.8, 123.4, 122.7, 112.8, 109.3, 57.0, 41.8, 28.7, 28.5 ppm; **LRMS (+ ESI)** m/z: 385 ([M + Na]^+^, 100), **(-ESI)** m/z: 361 ([M-H]^−^, 100); **HRMS (+ ESI)** Calc. for C_20_H_19_N_4_O_3_ [M + H]^+^: 363.14517, found 363.14450; **HPLC** R_T_ = 8.41 min, 99.47% (254 nm). Matched previously reported characterisation data [24].

#### 3-(2-chloro-7-methylquinolin-3-yl)-1-(4-hydroxyphenyl)prop-2-en-1-one (**13**)

To a solution of 2-chloro-7-methylquinoline-3-carbaldehyde (205 mg, 0.99 mmol) and 4-hydroxyacetophenone (138 mg, 1.01 mmol) in EtOH (2 mL) was added aq. NaOH (2.5 M, 0.8 mL, 1.99 mmol). The reaction was stirred at rt for 8 h. The resulting precipitate was filtered, washed with EtOH, and dried to obtain compound **13** as a white powder (255 mg, 79%) following purification by flash chromatography (0–100% v/v EtOAc in Hex). ^**1**^**H NMR** (500 MHz, DMSO-*d*_*6*_) *δ* 9.17 (s, 1 H), 8.12 (d, *J* = 8.8 Hz, 2 H), 8.09 (s, 1 H), 8.00 (s, 1 H), 7.96 (d, *J* = 8.7 Hz, 1 H), 7.77 (s, 1 H), 7.56 (dd, *J* = 8.4, 1.6 Hz, 1 H), 6.93 (d, *J* = 8.7 Hz, 2 H), 2.55 (s, 3 H) ppm; ^**13**^**C NMR** (126 MHz, DMSO-*d*_*6*_) *δ* 186.5, 162.6, 149.7, 147.4, 142.5, 137.1, 136.6, 131.4, 130.1, 128.7, 128.2, 126.7, 126.3, 126.0, 125.1, 115.5, 21.6 ppm; **LRMS (-ESI)** m/z: 322/324 ([M-H]^−^, 100); **HRMS (+ ESI)** Calc. for C_19_H_14_ClNNaO_2_ [M + Na]^+^: 346.06053/348.05758, found 346.06053/348.05760.

#### 1-(4-(benzyloxy)phenyl)-3-(2-chloro-7-methylquinolin-3-yl)prop-2-en-1-one (**16**)

To a suspended solution of **13** (50.1 mg, 0.15 mmol) and NaH (60% dispersion in mineral oil, 7.2 mg, 0.18 mmol) in DMF/THF (2:1 v/v, 1.5 mL) at 0 °C, was added benzyl bromide (25 µL, 0.21 mmol). The reaction mixture was brought to rt, with stirring over 3 h. The solution was cooled to 0 °C and quenched with 1 M HCl (1 mL) followed by addition of H_2_O (3 mL) and EtOAc (10 mL). The layers were separated, and the aqueous layer (pH > 4) was extracted with EtOAc (3 × 25 mL/mmol). The combined organic layers were washed with brine (1 × 25 mL/mmol), dried with MgSO_4_ and concentrated *in vacuo*. Compound **16** was isolated as a white solid (57.4 mg, 90%) following purification by flash chromatography (0–30% v/v EtOAc in Hex). ^**1**^**H NMR** (500 MHz, CDCl_3_) *δ* 8.44 (s, 1 H), 8.18 (d, *J* = 15.6 Hz, 1 H), 8.10–8.05 (m, 2 H), 7.81 (s, 1 H), 7.77 (d, *J* = 8.3 Hz, 1 H), 7.60 (d, *J* = 15.7 Hz, 1 H), 7.47–7.39 (m, 6 H), 7.38–7.33 (m, 1 H), 7.11–7.06 (m, 2 H), 5.17 (s, 2 H), 2.58 (s, 3 H) ppm; ^**13**^**C NMR** (126 MHz, CDCl_3_) *δ* 188.3, 163.0, 150.6, 148.3, 142.6, 139.0, 136.3, 136.0, 131.2, 131.0, 130.1, 128.9, 128.5, 127.8, 127.7, 127.7, 127.5, 126.0, 125.3, 115.0, 70.4, 22.3 ppm; **LRMS (+ APCI)** m/z: 414/416 ([M + H]^+^, 50); **HRMS (+ ESI)** Calc. for C_26_H_20_ClNNaO_2_ [M + Na]^+^: 436.10748/438.10453, found 436.10742/438.10448.

#### 4-(3-(4-(benzyloxy)phenyl)-5-(2-chloro-7-methylquinolin-3-yl)-4,5-dihydro-1*H*-pyrazol-1-yl)-4-oxobutanoic acid (**15**)

To a solution of **16** (49.8 mg, 0.12 mmol) in EtOH (1 mL) was added hydrazine monohydrate (40 µL, 0.82 mmol). The reaction was stirred at 80 °C for 2 h. Following consumption of starting material as indicated by TLC. The reaction mixture was diluted with H_2_O (3 mL) and EtOAc (10 mL). The layers were separated, and the aqueous layer (pH > 8) was extracted with EtOAc (3 × 25 mL/mmol). The combined organic layers were washed with brine (1 × 25 mL/mmol), dried with MgSO_4_ and concentrated *in vacuo* to yield the dihydropyrazole analogue (**17**). The residue was dissolved in CHCl_3_ (2 mL) and treated with succinic anhydride (26.7 mg, 0.27 mmol). The reaction was stirred at reflux 4 h. The precipitate was filtered and washed with EtOAc to yield compound **15** as a grey solid (50 mg, 81% over two steps). ^**1**^**H NMR** (500 MHz, DMSO-*d*_*6*_) *δ* 8.01 (s, 1 H), 7.89 (d, *J* = 8.4 Hz, 1 H), 7.76–7.71 (m, 3 H), 7.47 (dd, *J* = 8.4, 1.7 Hz, 1 H), 7.45 (d, *J* = 1.7 Hz, 1 H), 7.44 (s, 1 H), 7.38 (dd, *J* = 8.4, 6.6 Hz, 2 H), 7.35–7.30 (m, 1 H), 7.09 (d, *J* = 8.9 Hz, 2 H), 5.80 (dd, *J* = 11.9, 5.0 Hz, 1 H), 5.16 (s, 2 H), 4.06–3.94 (m, 1 H), 3.31–3.20 (m, 2 H) (overlapping with H_2_O peak), 2.89 (dt, *J* = 17.2, 6.4 Hz, 1 H), 2.65–2.52 (m, 2 H), 2.51 (s, 3 H) (overlapped with solvent peak) ppm; ^**13**^**C NMR** (126 MHz, DMSO-*d*_*6*_) *δ* 173.9, 169.1, 160.1, 155.2, 154.3, 148.0, 146.5, 140.9, 136.7, 131.5, 129.5, 128.4, 127.9, 127.8, 127.7, 126.3, 125.1, 123.6, 118.1, 115.1, 79.2, 69.3, 40.8, 28.6, 28.6, 21.4 ppm; **LRMS (+ ESI)** m/z: 550/552 ([M + Na]^+^, 100); **HRMS (+ ESI)** Calc. for C_30_H_26_ClN_3_NaO_4_ [M + Na]^+^: 550.15040/552.14745, found 550.15050/552.14756.

#### 4-(5-(2-chloro-7-methylquinolin-3-yl)-3-(4-hydroxyphenyl)-4,5-dihydro-1*H*-pyrazol-1-yl)-4-oxobutanoic acid diTFA salt (**4**)

A suspension of **15** (50 mg, 94.7 µmol) in CH_2_Cl_2_ (1 mL) was treated with BBr_3_ (1 M in CH_2_Cl_2_, 190 µL, 189 µmol) at 0 °C before stirring at rt for 1 h. Solvent was removed under a stream of N_2_. The reaction was taken up in CH_2_Cl_2_ and concentrated 2 more times. Compound **15** was isolated as the di-TFA salt (10.9 mg, 17%) following purification by reverse phase chromatography (0–100% v/v CH_3_CN in H_2_O, 0.1% TFA). ^**1**^**H NMR** (400 MHz, DMSO-*d*_*6*_) *δ* 12.12 (s, 1 H), 9.99 (s, 1 H), 8.00 (s, 1 H), 7.88 (d, *J* = 8.3 Hz, 1 H), 7.74 (s, 1 H), 7.63 (d, *J* = 8.6 Hz, 2 H), 7.47 (dd, *J* = 8.4, 1.7 Hz, 1 H), 6.82 (d, *J* = 8.8 Hz, 2 H), 5.78 (dd, *J* = 11.8, 4.9 Hz, 1 H), 3.96 (dd, *J* = 17.9, 11.9 Hz, 1 H), 3.29–3.19 (m, 2 H), 2.89 (dt, *J* = 17.0, 6.3 Hz, 1 H), 2.59–2.52 (m, 2 H), 2.51 (s, 3 H) (overlapped with solvent peak) ppm; ^**19**^**F NMR** (376 MHz, DMSO-*d*_*6*_) *δ* -73.4 ppm; ^**13**^**C NMR** (101 MHz, DMSO-*d*_*6*_) *δ* 173.9, 168.9, 159.6, 154.5, 148.0, 146.5, 140.8, 134.8, 131.5, 129.5, 128.5, 127.7, 126.3, 125.0, 121.7, 115.5, 57.1, 40.8, 28.5, 28.5, 21.3 ppm; **LRMS (+ ESI)** m/z: 460/462 ([M + Na]^+^, 100), **(-ESI)** m/z: 436/438 ([M-H]^−^, 100); **HRMS (+ ESI)** Calc. for C_23_H_20_ClN_3_NaO_4_ [M + Na]^+^: 460.10345/462.10050, found 460.10356/462.10072; **HPLC** R_T_ = 20.72 min, 95.10% (254 nm). Matched previously reported characterisation data [24].

### In silico

#### Protein preparation and refinement of the P2Y6R template homology model

The P2Y6R templated homology model was retrieved from Zhu et al. (2023) [23]. Utilising the Protein Preparation Wizard embedded in Maestro v13.9 the protein was prepared for further analysis. This process includes assigning bond orders, adding hydrogens atoms, and creating zero order bonds to metals and disulphide bonds. Additionally, missing side chains were filled in using the PRIME feature and heteroatom states between pH 7 ± 2 were generated with Epik [52–54]. The hydrogen bond network was then optimised through the PROPKA function, using sample water orientations and the protonation state for each heteroatom as defined at pH 7. The protein was further optimised by removing any water molecules involved in hydrogen bonding further than 5Å from a heteroatom. Finally, the total protein structure was energy minimised using the OPLS4 force field, and heavy atoms converged to a RMSD of 0.3 Å [55].

#### Protein preparation and refinement of the P2Y6R AlphaFold homology model

The human P2Y6R amino acid sequence (uniprotKB: Q15077) was retrieved from UniProt and submitted as a full receptor into AlphaFold2. The prediction was generated using localColabfold. The protein was then prepared for further analysis utilizing the Protein Preparation Wizard in Maestro v13.9, via the methodology described.

#### Ligand preparation

Compound structures were imported directly into the Maestro software from ChemDraw v23.1. All ligands were prepared using the LigPrep module to generate all potential ionisation states and tautomers at pH 7 ± 2 [52, 53, 55]. Confgen was also used to generate up to 64 different conformations for each ligand [54]. By generating the conformations bias introduced by the configuration of the starting molecule is removed [54]. ConfGen provides a good approximation of the bioactive geometry of a ligand by rejecting high energy or inactive conformers and identifying local torsional minima [54].

#### Receptor grid generation

The Receptor Grid Generation tool in Glide v9.1 was used to characterise the binding site for the docking studies [56]. The first binding site was defined by a 20 Å bounding box centred at the co-crystalised ligand within the allosteric site of chain A. A Coulomb-van der Waals scaling factor of 1.0 for receptor van-der Waals radii was applied to protein atoms with a partial charge of less than 0.25 e and a similar factor of 0.8 was applied to ligand atoms with a partial charge cutoff of less than 0.15 e. Rotations of hydroxyl and thiol groups were not allowed.

#### Rigid target docking of P2Y6R ligands

Compound structures were prepared in accordance with the methodology described prior. Similarly, a receptor grid was defined by a 20 Å bounding box centred at the co-crystalised ligand and residues listed by Zhu et al. (2023) and Zhao et al. (2024) for the template and AlphaFold homology models respectively [23, 24]. All other parameters were kept consistent. The ligands (**1** and **2**) were docked into the respective receptor grides with Glide v9.1. All docking studies were carried out using the Extra Precision (XP) scoring function and identical parameters. Docking experiments were conducted with the OPLS4 and OPLS2005 force fields for both homology model types. Compounds were additionally sourced from the original paper to provide further validation [23].

#### Induced-fit docking

Compound 5 − **1**, reported by Zhu et al. (2023), was docked into the receptor with Induced Fit Docking default settings [23, 57]. Calculations were made using standard protocol and OPLS4 force field [55]. Initial ligand conformations for each compound were generated from the top ranked pose from the initial docking studies. Binding sites were defined by a 20 Å bounding box centred at the co-crystalised ligand within the P2Y6R active site. Receptor van der Waals scaling and Ligand van der Waals scaling was set to 0.50. Residues within 20 Å of ligand poses were refined, with optimisation of side chains. Ligands were redocked with Extra Precision (XP) to refine binding energy estimates [56, 58]. All ligands were docked with flexible states to allow sampling of the effort of nitrogen interconversion, changing ring conformations and non-planar amide functional groups were penalised [58].

### In vitro

#### P2Y6R Ca^2+^ flux assay

The 132-1N1 cell line stably expressing the human P2Y6 receptor was purchased from PerkinElmer (Product No. ES-292-C, Lot No. 1757915). 132-1N1-hP2Y6R, CHO-WT and/or HEK-WT cells were detached using trypsin/EDTA, collected via centrifuge (150 g, 5 min) and resuspended in culture media. Resuspended cells were plated at 2.0 × 10^4^ cells/well into 96-well black-walled CellBIND plates (Corning) and incubated for 24 h (37 °C, 5% CO_2_). To measure the downstream accumulation of Ca^2+^ following P2Y6R activation, the Ca^2+^ flux Fluo-4 NW (Invitrogen) was utilised as per the manufacturer’s protocol. After incubation, culture media was replaced with 80 µL dye solution containing Fluo-4 NW (Invitrogen), probenecid (5 mM, Invitrogen) dissolved in modified Hanks’ Buffered Saline Solution assay buffer (KCl 5.37 mM, KH_2_PO_4_ 0.44 mM, NaCl 137 mM, NaH_2_PO_4_ 0.38 mM, glucose 5.5 mM, CaCl_2_ 1.26 mM, HEPES 20 mM, MgCl_2_ 0.5 mM, MgSO_4_ 0.41 mM, NaHCO_3_ 4.17 mM, pH 7.4) and incubated for 0.5 h at 37 °C. Working antagonist compound concentrations ranged from 0.001 µM – 100 µM with the addition of agonist UDP (0.075 µM or 10 µM) and treated onto cells either with, or without, a 30 min pre-incubation in antagonists. Fluorescence (λ_Ex_ = 494 nm, λ_Em_ = 516 nm) was recorded for 31 s for a baseline and then 20 µL was injected onto cells at 3 µLs^-1^ with fluorescence recorded for a further 150 s using a Flexstation 3 Multi-Mode Microplate Reader (Molecular Devices) using the same instrument settings as outlined in Table 1.

**Table 1:** Flexstation 3 Multi-Mode Microplate Reader settings used for Ca^2+^ Flux assay

Flex	Fluorescence	PMT and Optics	More Settings	Fluidic Settings
Time: 120 s	Ex: 494	Medium	Shake off	T#1 @ 30 s
Interval: 2.0 s Reads: 61	Auto Cutoff: 515 Em: 516	6 Flashes/read Read from Bottom	Calibration On	H = 80 µL *R* = 2 V = 20 µL Trit: C(V = 20 µL, C = 1)

Collected fluorescence data was normalized by subtracting each fluorescence signal after 31s by the average signal of 0–31 s before injection of agonist (F_0_) for each well. Normalised antagonist data was analysed for area under the curve (AUC) using GraphPad 10.0 (Prism). Mean Total peak area of Ca^2+^ signal was used to compare differences to mean-agonist only conditions, using a one-way analysis of variance (ANOVA) with a Dunnett’s post-hoc test. Mean total peak area was converted to %UDP activity by dividing mean total peak area by mean-agonist only conditions and analysed using a 4-parameter sigmoidal dose-response (antagonist) fit in GraphPad 10.0.

## Supplementary Information

Below is the link to the electronic supplementary material.

Supplementary Material 1

## Data Availability

No datasets were generated or analysed during the current study.
