# Supplementary material for: Investigation of reported small molecule P2Y6 receptor antagonists
Source: Med Chem Res. 2026 Jul 11;35(10):2055–72. doi: 10.1007/s00044-026-03591-w (PMC13669135; doi:10.1007/s00044-026-03591-w)
Supplement: Supplementary file 1 [file 44_2026_3591_MOESM1_ESM.docx]

**Investigation of reported small molecule P2Y6 receptor antagonists.**

Chianna Dane^*,1^, André D.J. McKenzie^*,1^, Andrew P. Montgomery^1^, Jonathan Du^2^, Eryn L. Werry^1^, Michael Kassiou^**,1^

* Authors contributed equally to this work

** Corresponding author. Email: [michael.kassiou@sydney.edu.au](mailto:michael.kassiou@sydney.edu.au)

^1^School of Chemistry, Faculty of Science, University of Sydney, Sydney, NSW 2006, Australia

^2^Sydney Pharmacy School, Faculty of Medicine and Health, University of Sydney, Sydney, NSW 2006, Australia

**Supplementary Information:**

- Structural Alignment of Homology Models	page 2
- Docking Scores	page 3
- Comparison of *In Vitro* Assays	page 4
- MRS2578 Synthetic Procedure	page 5

**Supplementary 1**: Structural Alignment of Homology Models

Protein preparation and refinement was conducted on both the template and AF homology model as per the methodology outlined in the paper. Disordered *C-* and *N*-terminal loops were removed from both proteins, with only the protein backbone and not ligand classified as reference residues. Residues were then aligned using the same atom specification language as reference residues. The alignment score was found to be 0.177, with an RMSD value of 1.8 Å.

*Figure 1.* Structural alignment analysis of P2Y6 template (red) and AF (green) homology models. A) Alignment of protein backbone with disordered terminal loops removed produced a RMSD value of 1.8 Å. B) Defined ligand bonding domain by the reported template homology model with Zhu *et al.* hit molecule, compound **1a**, docked in the published PDB file.

**Supplementary 2:** *In silico* hP2Y6R induced-fit docking (IFD) and rigid-target docking (RTD) scores of reference compounds. A more negative score denotes a stronger binding affinity

| Compound | Template Homology | | | AlphaFold |
| --- | --- | --- | --- | --- |
|  | IFD Score (kcal/mol) | RTD Score (kcal/mol) | | RTD Score (kcal/mol) |
|  | OPLS4 | OPLS4 | OPLS2005 | OPLS4 |
| *5*-1 | -7.96 | -2.22 | -2.22 | -4.63 |
| *6*-1 |  | -2.31 | -2.31 | -4.90 |
| 3 |  | -2.53 | -4.17 | -5.10 |
| (*R*)-2 |  | -6.39 | -6.39 | -8.20 |
| (*S*)-2 |  | -6.04 | -6.04 | -9.21 |
| (*R*)-4 |  | -4.44 | -4.44 | -9.39 |
| (*S*)-4 |  | -4.81 | -4.83 | -8.66 |

**Supplementary 3:** Summary of experimental conditions of published or referenced P2Y6R antagonist assays.

| Pre-Incubation (min) | 0 | 30 | 30 | N/R^a^ | 45 | N/R^a^ |  | ^a^N/R = not reported. 132-1N1: human astrocytoma cells; HEK: human embryonic kidney cells; CHO: Chinese hamster ovary cells. |
| --- | --- | --- | --- | --- | --- | --- | --- | --- |
| UDP Exposure | 1 – 3 min | 12 h | 12 h | 1 – 3 min | 1 – 3 min | N/R^b^ |  |  |
| [UDP] nM | 75 | 10000 | 10000 | N/R^b^ | 100 | 10 |  |  |
| Detection | Fluorescence | Absorbance | Absorbance | Fluorescence | Fluorescence | Fluorimetry |  |  |
| Kit (Company) | Fluo-4 NW  (Invitrogen) | IP_3_ ELISA  (Elabscience) | IP_3_ ELISA  (Elabscience) | FLIPR Calcium 6  (Molecular Devices) | FLIPR Calcium 6  (Molecular Devices) | Proprietary |  |  |
| Assay | Ca^2+^ Flux | IP_3_ accumulation | IP_3_ accumulation | Ca^2+^ Flux | Ca^2+^ Flux | Ca^2+^ Flux |  |  |
| Cell Type | 132-1N1-hP2Y6R | HEK293-hP2Y6R | HEK293-hP2Y6R | CHO-hP2Y6R | 132-1N1-hP2Y6R | 132-1N1-hP2Y6R |  |  |
| Source |  | Li *et al.* (2022) | Zhu *et al.* (2023) | Zhao *et al.* (2024) | Oliva *et al*. (2024) | Eurofins Scientific |  |  |

**Supplementary 4:** MRS2578 Synthetic Procedure

*1,1'-(butane-1,4-diyl)bis(3-(3-nitrophenyl)thiourea)*

1,1′-Thiocarbonyldiimidazole (221 mg, 1.24 mmol) was added portionwise to a stirring solution of 3-nitroaniline (146 mg, 1.06 mmol) in CH_2_Cl_2_/THF (3 mL, 2:1 v/v). The reaction mixture was stirred at rt, until complete conversion of starting material as indicated by TLC. Butane-1,4-diamine (50 µL, 0.51 mmol) was diluted in THF (0.5 mL), and added dropwise over a 5 min period. Following consumption of starting material, as indicated by TLC, the reaction mixture was concentrated. Purification by reverse phase column chromatography (0 – 100% MeCN:H_2_O) yielded the title compound as a yellow solid (35 mg, 15%).

**^1^H NMR** (300 MHz, DMSO-*d_6_*) δ 9.98 (s, 2H), 8.61 (s, 2H), 8.20 (s, 2H), 7.90 (dd, *J* = 8.3, 1.5 Hz, 2H), 7.82 (d, *J* = 8.1 Hz, 2H), 7.57 (t, *J* = 8.1 Hz, 2H), 3.53 (s, 5H), 1.68 – 1.55 (m, 4H) ppm; **LRMS (-ESI)** m/z: 447 ([M-H]^-^, 100).

*1,1'-(butane-1,4-diyl)bis(3-(3-isothiocyanatophenyl)thiourea) –* **MRS2578**

4,4-Bipyridine (1 mg, 0.008 mmol) was added to a stirring solution of 1,1'-(butane-1,4-diyl)bis(3-(3-nitrophenyl)thiourea) (20 mg, 0.045 mmol) in DMF (1 mL) at 0 °C. B_2_(OH)_4_ (24 mg, 0.267 mmol) was then added portionwise over a 15 min period at 0 °C. The reaction was then stirred at rt for a further 15 min. Following consumption of starting material, as indicated by TLC, the reaction mixture was concentrated under a stream of nitrogen and carried through without further purification. The crude residue was redissolved in DMF (1 mL). 1,1′-Thiocarbonyldiimidazole (20.4 mg, 0.114 mmol) was added to the reaction mixture and stirred at rt until complete conversion of starting material as indicated by TLC. The title compound (3.2 mg, 15% over two steps) was isolated as an off-white powder following purification by reverse phase column chromatography (0 – 100% MeCN:H_2_O).

**^1^H NMR** (500 MHz, DMSO-*d_6_*) *δ* 9.66 (s, 2H), 7.99 (s, 2H), 7.64 (s, 2H), 7.40 – 7.33 (m, 4H), 7.17 – 7.10 (m, 2H), 3.50 (s, 4H), 1.61 – 1.54 (m, 4H) ppm; **^13^C NMR** (126 MHz, DMSO-*d_6_*) *δ* 180.3, 140.8, 133.9, 129.9, 121.9, 121.0, 119.3, 43.6, 26.0 ppm; **LRMS (+ESI)** m/z: 495 ([M+Na]^+^, 100), **(-ESI)** m/z: 471 ([M-H]^-^, 100); **HPLC** R_T_ = 27.18 min, 96.8% (254 nM). Characterisation data matches literature reference [Mamedova *et al*., 2004].

**^1^H NMR** (500 MHz, DMSO-*d_6_*):

**^13^C NMR** (126 MHz, DMSO-*d_6_*):

**HPLC** R_T_ (254 nM):

Mamedova LK, Joshi BV, Gao Z-G, et al. Diisothiocyanate derivatives as potent, insurmountable antagonists of P2Y6 nucleotide receptors. Biochemical Pharmacology. 2004;67:1763–70. https://doi.org/10.1016/j.bcp.2004.01.011.
